# Frailty, Physical Performance, and Molecular Changes in Older Adults Participating in Different Exercise Programs: A 12-Week Preference-Based Non-Randomized Study

**DOI:** 10.3390/nu18183052

**Published:** 2026-09-18

**Authors:** Jiahao Li, Huizhi Yang, Hefu Zhen, Zhiming Li, Jiali Lu, Huiping Yan, Chao Nie, Yifan Lu

**Affiliations:** 1 School of Sports Medicine and Rehabilitation, Beijing Sport University, Beijing 100084, China; 2 Shenzhen Key Laboratory of Neurogenomics, BGI-Shenzhen, Shenzhen 518120, China; 3 State Key Laboratory of Genome and Multi-Omics Technologies, BGI Research, Shenzhen 518083, China; 4 Key Laboratory of Sports and Physical Fitness of the Ministry of Education, Beijing Sport University, Beijing 100084, China

**Keywords:** frailty, exercise, older adults, targeted metabolomics, short-chain fatty acids, gut microbiome, healthy aging

## Abstract

Background: Multidimensional evidence on exercise participation and frailty in older adults is limited. Methods: This 12-week preference-based, non-randomized study included 145 participants (mean age 82.25 years): walking (*n* = 50), resistance training (*n* = 20), Traditional Chinese exercise (*n* = 23), and control (*n* = 52). Frailty was the primary outcome; physical-performance and molecular analyses were exploratory. Paired trajectories, baseline-stratified binary transitions, and adjusted associations were examined. Results: Among paired participants, pre-frailty/frailty prevalence changed from 70.0% to 60.0% with walking, 90.0% to 70.0% with resistance training, 50.0% to 63.6% with Traditional Chinese exercise (*n* = 22), and 50.0% to 69.2% in controls. Baseline status constrained opportunities for binary improvement or deterioration. All adjusted frailty risk-ratio confidence intervals included 1. Walking was associated with faster Timed Up and Go and Five-Yard Walk Test performance after correction across eight outcomes. Molecular changes also occurred in controls; intervention timing and potential laboratory-batch confounding limited between-group interpretation. No microbiome taxon survived multiplicity correction; small paired samples limited precision. Conclusions: Walking and resistance-training groups showed descriptively more favorable prevalence trajectories, but adjusted frailty comparisons remained inconclusive. These findings do not establish frailty prevention, modality superiority, or exercise-specific molecular mechanisms.

## 1. Introduction

Frailty is a prevalent geriatric syndrome characterized by reduced physiological reserve and increased vulnerability to adverse outcomes, including falls, hospitalization, and mortality [1,2]. With the rapid growth of the aging population, effective strategies to prevent or attenuate frailty are urgently needed. Physical activity has been linked to frailty-associated protein profiles [3], and exercise trials have reported improvements in frailty-related outcomes in older adults [4]. However, whether different exercise modalities exert distinct effects on frailty, particularly in older adults, remains unclear.

The biological processes linking exercise participation and frailty remain incompletely understood. Exercise omics research has characterized molecular responses in skeletal muscle [5,6]; combining clinical measures with circulating metabolite and gut microbial profiles may extend this approach to systemic variation in older adults. Prior molecular studies motivate multidimensional assessment [7], but circulating metabolite concentrations and microbial composition alone cannot establish the mechanisms through which exercise may influence frailty.

We examined 12-week changes in frailty among older adults participating in walking, resistance training, Traditional Chinese exercise, or a non-exercise control condition. Physical performance, circulating biomarkers, and gut microbiome profiles were assessed as exploratory outcomes. Baseline-adjusted comparisons were used to describe associations between program participation and follow-up outcomes.

## 2. Materials and Methods

### 2.1. Study Design and Participants

This study is reported with reference to the Transparent Reporting of Evaluations with Nonrandomized Designs (TREND) statement [8], given its non-randomized, preference-based intervention design. This was a 12-week non-randomized, preference-based intervention conducted in a senior care facility in Langfang, Hebei, China. Nursing-home residents aged ≥60 years who were able to walk independently were recruited. Participants were required to have low-to-moderate cardiovascular risk and provide written informed consent.

Exclusion criteria included recent major surgery or fractures, the use of antibiotics or probiotics within one month prior to sampling, gastrointestinal disorders, and severe chronic or psychiatric conditions that could limit exercise participation.

Participants were recruited through on-site enrollment following health education lectures held at the senior care facility.

Participant recruitment and the 12-week intervention and follow-up period were conducted between November 2022 and June 2023. The exercise programs were not conducted concurrently.

**Figure 1:**
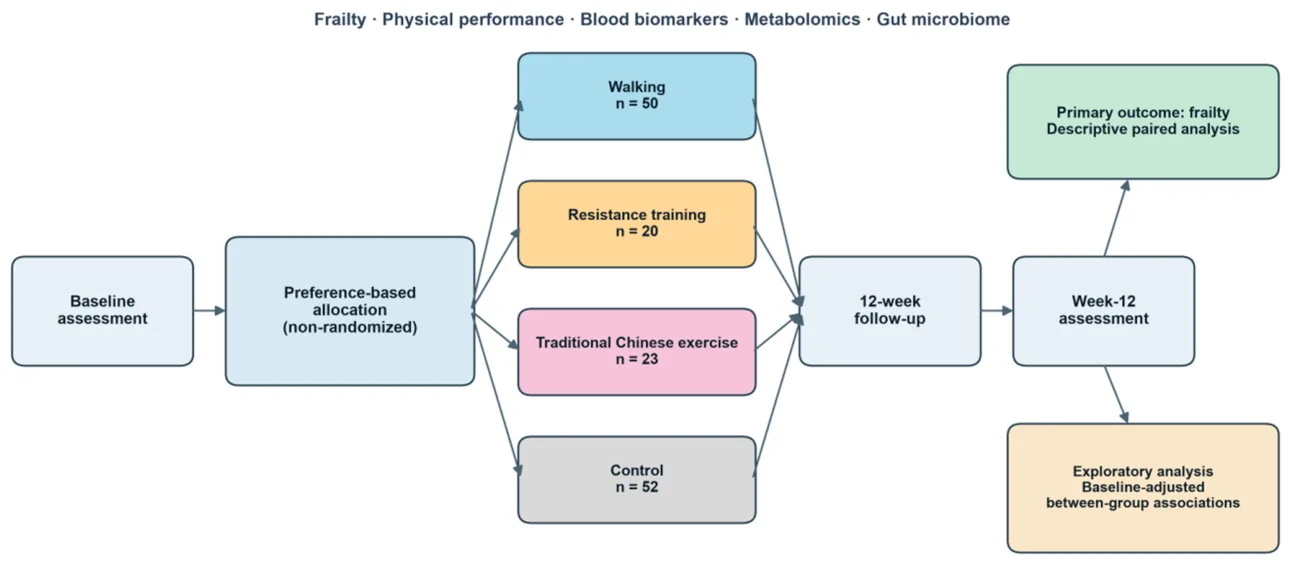
Study design and analytical framework.

Participants were allocated according to their preference to walking, resistance training, Traditional Chinese exercise, or a non-exercise control group. Assessments were conducted at baseline and after 12 weeks. Blood and stool samples were collected at both time points. The study design is shown in Figure 1. Exercise preference may be related to baseline function, health, prior activity, motivation, perceived capability, and expectations; allocation therefore did not ensure exchangeable groups.

No formal a priori sample size calculation was performed; given the recruitment challenges associated with this very old population (mean age 82 years), the sample size was determined pragmatically based on the number of eligible and willing participants during the recruitment period. Consequently, particularly in the smaller resistance-training and Traditional Chinese exercise groups, estimates may be imprecise and sensitive to a few observations. Confidence intervals are reported to describe uncertainty; no retrospective power calculation was undertaken.

### 2.2. Interventions

Participants in the walking group were prescribed a 12-week supervised walking program at an intensity of 40–60% heart rate reserve (HRR). Training intensity was progressively increased from 40–50% HRR during weeks 1–6 to 50–60% HRR during weeks 7–12. Sessions were conducted three times per week and consisted of 45 min of continuous walking, preceded by a 5 min warm-up and followed by a 5 min cool-down; heart rate was continuously monitored to guide the prescribed target intensity.

The resistance training group performed elastic band exercises using TheraBand (Hygenic Corporation, Akron, OH, USA) three times per week (Monday, Wednesday, and Friday). Each session comprised a 10 min warm-up, 40 min of resistance training, and a 10 min cool-down. Training targeted the major muscle groups of the upper and lower limbs (shoulder, elbow, knee, and ankle), with each exercise performed for 2–3 sets of 8–12 repetitions. Resistance level was individually adjusted every 4 weeks based on the estimated one-repetition maximum (1RM) and participant tolerance.

The Traditional Chinese exercise group practiced a structured Daoyin program comprising two standardized routines (Yangsheng Zhujigong and Jianpi Qiangji Daoyin) over 12 weeks. During weeks 1–4, participants received supervised training focused on standardized movement learning (1 h/session, 3–4 sessions/week); during weeks 5–12, participants continued practicing the routines at the same frequency and duration under supervision.

The control group received health education lectures at a frequency and duration matched to the intervention groups, covering healthy aging, physical activity guidance, and chronic disease management; no structured exercise intervention was provided.

The prescribed schedule corresponded to 36 walking sessions of 55 min, 36 resistance-training sessions of 60 min, and 36–48 Traditional Chinese exercise sessions of 60 min over 12 weeks. Members of the intervention team were based at the nursing home, notified participants before each scheduled session, and supervised the exercise sessions. Attendance was not systematically documented; participant-level attendance percentages, completed-session counts, and achieved exercise intensity or volume could not be reliably quantified. An attendance threshold of 80% had been planned, but its implementation cannot be verified from the available records. The final analysis population comprised participants who completed the week-12 assessment, with outcome-specific complete-case eligibility. Completion of follow-up does not establish completion of all prescribed sessions. The session totals above represent the prescribed schedule.

Adverse events were assessed through monthly inquiries by the research team and spontaneous reports from participants at any time during the study.

### 2.3. Sample Collection and Measurements

Frailty status was the primary outcome and was assessed with a study-specific operationalization of the five-domain Fried/CHS phenotype [9], using the scoring criteria described below. Analyses used the recorded component scores; a complete reassessment from the original questionnaire items was not possible for all domains. One point was assigned for each of the following: (1) unintentional weight loss >3 kg during the preceding 6 months, unrelated to dieting or exercise; (2) low handgrip strength, defined using sex- and BMI-specific thresholds (men: ≤29 kg for BMI ≤ 24 kg/m^2^, ≤30 kg for 24 < BMI ≤ 28 kg/m^2^, and ≤32 kg for BMI > 28.0 kg/m^2^; women: ≤17 kg for BMI ≤ 23 kg/m^2^, ≤17.3 kg for 23 < BMI ≤ 26 kg/m^2^, ≤18 kg for 26 < BMI ≤ 29 kg/m^2^, and ≤21 kg for BMI > 29.0 kg/m^2^); (3) exhaustion, defined as reporting on more than 3 days during the preceding week that everything was an effort or that the participant could not get going; (4) slowness on the usual-pace Five-Yard Walk Test, defined as ≥7 s for men ≤ 173 cm or women ≤ 159 cm and ≥6 s for taller participants; and (5) low physical activity, defined as estimated weekly energy expenditure <383 kcal for men and < 270 kcal for women. The recorded low-activity component was used; the activity-to-energy conversion could not be verified from the available scoring workbook. Components were coded 1 when present and 0 when absent. Total scores ranged from 0 to 5 and were categorized as robust (0), pre-frail (1–2), or frail (3–5). If any of the five component values was missing, the total and categorical frailty status for that assessment were set to missing; missing components were not coded as absent. For the primary paired analysis, pre-frail and frail participants were combined and compared with robust participants. The transition analyses therefore distinguish robust from combined pre-frail/frail status, not changes between pre-frailty and frailty. Physical-performance measures and biological markers were secondary exploratory outcomes; they were not treated as confirmatory efficacy endpoints.

#### 2.3.1. Baseline and Anthropometric Assessment

Baseline demographic and clinical data were collected by trained investigators using standardized face-to-face interviews. Anthropometric and body composition measurements were conducted using standardized procedures. Height was measured with a calibrated stadiometer, with participants barefoot, standing upright, and the head in the Frankfort plane; measurements were recorded to 0.1 cm. Body composition was assessed using a multi-frequency bioelectrical impedance analyzer after participants had rested for 10 min, yielding their body weight, skeletal muscle mass, body fat percentage, and body mass index (BMI). Nutritional status was assessed using the Mini Nutritional Assessment-Short Form (MNA-SF), and cognitive function was assessed using the Mini-Mental State Examination (MMSE).

#### 2.3.2. Physical Performance Assessment

Physical performance was evaluated using eight standardized tests: handgrip strength (HGS; measured with a digital dynamometer, maximum value of two trials per hand); the Timed Up and Go (TUG; time to stand, walk 3 m, turn, return, and sit, using best of two trials); the Short Physical Performance Battery (SPPB; chair stand, balance, and 4 m gait speed, 0–12 score); the Berg Balance Scale (BBS; a 14-item balance assessment, 0–56 score); the Six-Minute Walk Test (6MWT; total distance walked in 6 min along a 20 m corridor); the Chair Sit-and-Reach Test (maximum reach distance, cm); the Five-Yard Walk Test (5YWT; usual gait time over 5 yards); and the Back Scratch Test (shoulder flexibility measured as fingertip distance, cm).

#### 2.3.3. Blood Biomarkers and Targeted Metabolomics

Fasting venous blood samples (≥10 h fasting) were collected in the morning into ethylenediaminetetraacetic acid (EDTA) tubes. A separate aliquot of EDTA-anticoagulated whole blood was sent to the hospital clinical laboratory for red blood cell, hemoglobin, and lymphocyte counts using an automated hematology analyzer under the laboratory’s routine internal quality-control procedures. The remaining blood was centrifuged at 3000 rpm for 5–10 min at 4 °C, and plasma was stored at −80 °C until targeted metabolomic analysis by BGI Genomics. Targeted metabolites, including short-chain fatty acids (SCFAs), amino acids, steroid-related metabolites, and vitamins, were measured using liquid chromatography–tandem mass spectrometry (LC–MS/MS, (LC–MS/MS; Transcend II, Thermo Fisher Scientific, Franklin, MA, USA; SCIEX 5500, SCIEX, Framingham, MA, USA)) under the provider’s standardized analytical and quality-control procedures. For SCFAs, plasma samples were thawed stepwise (−20 °C to 4 °C), subjected to protein precipitation with methanol/acetonitrile (2:1), centrifuged (20,000× *g*, 15 min), derivatized with 3-nitrophenylhydrazine and 1-ethyl-3-(3-dimethylaminopropyl)carbodiimide/pyridine at 40 °C for 30 min, and spiked with internal standards before analysis (10 μL injection volume). For amino acids, samples were precipitated with 10% sulfosalicylic acid, vortexed, diluted, and centrifuged (4000 rpm, 20 min, 4 °C), and the supernatants were analyzed by LC–MS/MS. Calibration standards, internal standards, and laboratory quality-control samples were used as applicable to the corresponding assay panel.

#### 2.3.4. Gut Microbiome Metagenomic Analysis

For gut microbiome profiling, fecal samples were self-collected using standardized kits (BGI, China); approximately 0.5 g of stool was stored in stabilization buffer at room temperature (≤2 weeks) before transfer to −80 °C. DNA was extracted using InhibitEX buffer and Proteinase K digestion followed by silica-column purification, and libraries (300–400 bp) were sequenced on the DNBSEQ platform (BGI). Raw reads were quality-filtered by removing reads with >10% ambiguous bases, adapter-contaminated reads (>15 bp overlap), low-quality reads (Q20 < 60%), and host-derived reads (identified using SOAP2, identity threshold 90%).

Physical-performance assessors were not informed of participants’ intervention-group assignments during outcome assessment. Laboratory personnel received coded specimens and performed hematological, metabolomic, and microbiome testing without access to intervention-group information.

### 2.4. Data Processing and Statistical Analysis

**Figure 4:**
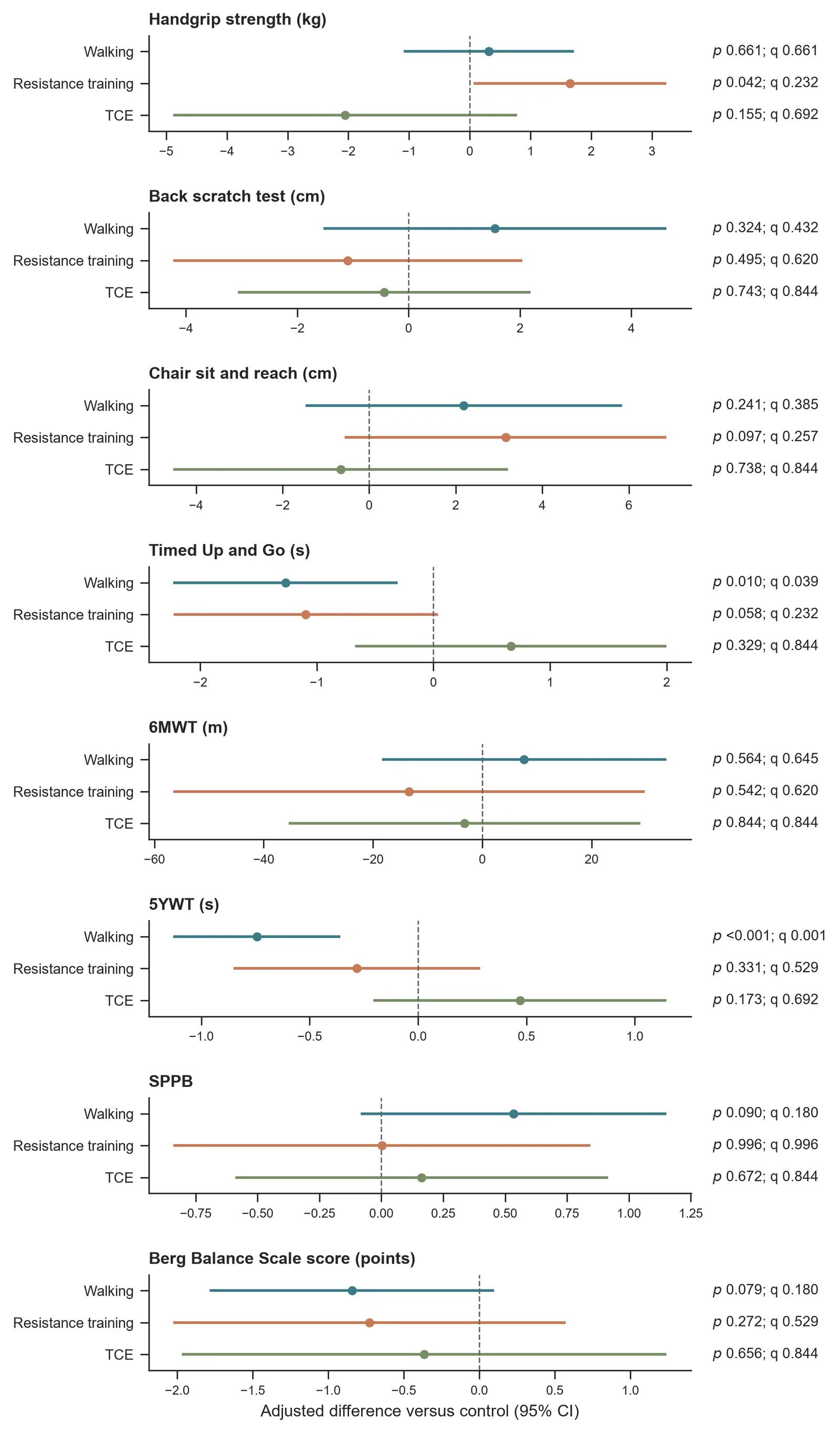
Baseline-adjusted between-group differences in eight physical-performance outcomes at week 12. Points show adjusted mean differences versus the control, and lines show 95% confidence intervals. *p* values are nominal; q values were calculated using Benjamini–Hochberg correction across all eight outcomes separately for each contrast. TCE, Traditional Chinese exercise; 5YWT, Five-Yard Walk Test.

Statistical analyses were conducted on an outcome-specific complete-case basis. Participants contributed to a paired analysis when both baseline and week-12 values were available for the relevant outcome. Continuous variables are presented as the mean ± standard deviation or median (interquartile range), as appropriate. Within-group continuous changes were evaluated using paired t-tests, with Wilcoxon signed-rank tests used as distribution-robust sensitivity analyses. Changes in the paired binary frailty classification were evaluated using exact McNemar tests. Within-group analyses were considered descriptive; differences between their *p* values were not interpreted as evidence of differences between interventions. Paired physical-performance effects are expressed as mean week-12 minus baseline differences in the original units, with two-sided 95% t-based confidence intervals using the standard deviation of the individual paired differences. These intervals are descriptive and not multiplicity-adjusted. Table 1, Tables S2 and S4 and Figure 4 were generated from the same final analysis dataset. Table 1 uses available baseline measurements; paired summaries require both time points, and adjusted models additionally require complete baseline covariates.

In a post hoc descriptive analysis, binary frailty transitions were stratified by baseline status. Among initially robust participants, deterioration was transition to pre-frailty/frailty; among initially pre-frail/frail participants, improvement reflected the transition to a robust status. Unchanged binary status was reported separately in each stratum. Transition proportions use the number in the respective baseline stratum as the denominator, with 95% Wilson confidence intervals. These summaries are not adjusted causal contrasts and cannot distinguish pre-frailty from frailty.

For the eight physical-performance outcomes, exploratory between-group comparisons used analysis of covariance with the week-12 value as the dependent variable and adjustment for the baseline value, age, sex, and body mass index (BMI); HC3 robust standard errors were used. Benjamini–Hochberg false-discovery-rate correction was applied across all eight outcomes separately for each intervention-versus-control contrast, including 5YWT, because analyzable paired and model data were available. For pre-frailty/frailty, modified Poisson regression with robust standard errors estimated adjusted risk ratios at week 12 after adjustment for baseline frailty, age, sex, and BMI. Covariates were selected for their clinical and biological relevance, rather than baseline significance tests, stepwise selection, or screening against the observed results. The baseline value of each outcome accounted for differences in the starting status and its relationship with the follow-up value. Age and sex were included because they are relevant to frailty, physical performance, and biological measurements and may also influence program preference. BMI represented body size, which may be related to program selection and the outcomes. A limited adjustment set was used given the small samples in several groups. Each adjusted model included the corresponding baseline outcome, baseline age, sex, and BMI; post-intervention covariates were not included. This set cannot account for all determinants of program choice and outcomes, so the estimates were interpreted as adjusted associations rather than causal effects.

Participants resided in the same nursing-home setting. Meals were routinely provided by the facility and were reported to remain generally unchanged during the 12-week study; medication regimens and usual daily activities were also intended to remain consistent with each participant’s routine. The shared residential setting may have reduced environmental variation; however, quantitative longitudinal exposure records were not available for analysis, so the degree of dietary, medication, and activity stability could not be assessed quantitatively. Baseline medication-count and individual comorbidity questionnaire fields were available for 140/145 participants, MMSE data for 142/145, and MNA-SF data for 145/145 (Supplementary Table S10). MNA-SF and MMSE were not included in the core adjustment set. Quantified protein/energy intake, supplement use, medication changes, and prior structured exercise exposure were not available for adjustment. Although a low-activity component was recorded for frailty scoring, it did not provide a verified, harmonized continuous measure of baseline activity for covariate adjustment.

For blood and targeted metabolomic variables, observed paired measurements were analyzed without single-imputation substitution. Positive concentrations were log2 transformed, and paired log-ratios were tested within each group; Benjamini–Hochberg correction was applied within each molecular family defined for the analysis. Direct between-group molecular models adjusted for baseline log2 concentration, age, sex, and BMI were considered sensitivity analyses because group and laboratory-batch effects could not be fully separated. For the microbiome, taxa present in at least 10% of samples were retained, relative abundances were centered-log-ratio transformed, and paired taxon-level changes were corrected using the Benjamini–Hochberg procedure. Shannon diversity was tested using paired Wilcoxon tests, and Bray–Curtis beta diversity was evaluated using within-participant label-permutation permutational multivariate analysis of variance (PERMANOVA). These corrections apply to the stated analysis families, not to the study as a whole, and do not remove confounding, selection bias, or technical measurement variation.

Paired microbiome availability was audited by linking clinical sample identifiers to profiles at both time points. Baseline age, sex, BMI, and binary frailty were summarized separately for participants with and without paired profiles within each arm. Missing microbiome profiles were primarily attributable to participants not providing a stool sample. No missingness-at-random assumption was established, and missing molecular profiles were not imputed.

The primary interpretation emphasized effect estimates, 95% confidence intervals, multiplicity-adjusted results, and consistency with changes in the control condition. All analyses were performed using Python 3.12 with SciPy, statsmodels, and scikit-learn; figures were generated using Matplotlib: 3.11.1 and Seaborn: 0.13.2.

## 3. Results

**Figure 2:**
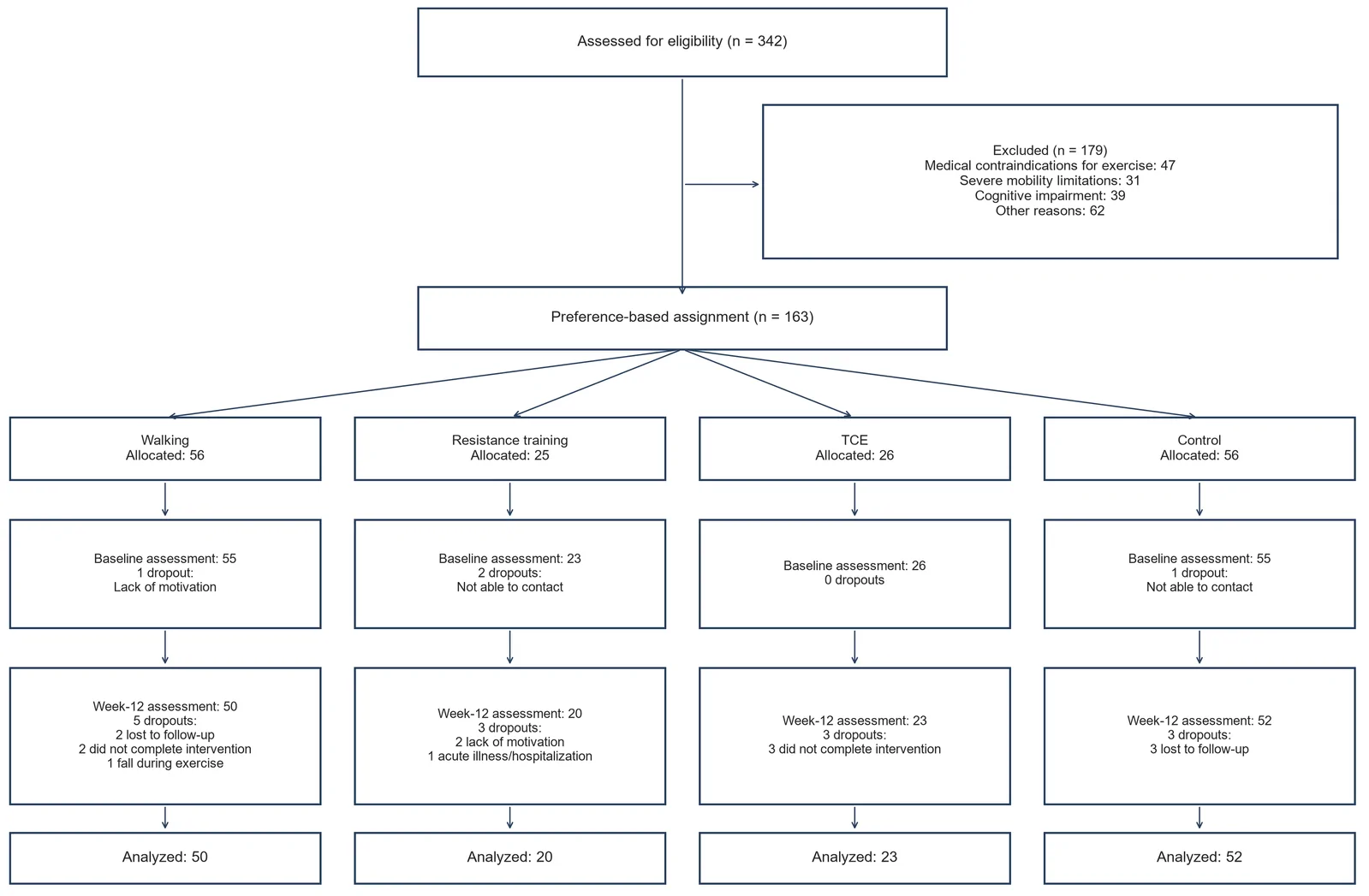
Participant flow. Of 163 allocated participants, 159 underwent baseline assessment, and 145 completed week-12 assessment. Analyses included participants with available measurements for each outcome. Intervention noncompletion and other recorded withdrawal reasons are shown separately. Session-level attendance was not systematically recorded. TCE, Traditional Chinese exercise.

A total of 342 older adults were assessed for eligibility, of whom 163 entered the study and 145 were included in the final analytic sample: walking, *n* = 50; resistance training, *n* = 20; Traditional Chinese exercise, *n* = 23; and control, *n* = 52 (Figure 2). The sample included 54 men and 91 women, with a mean age of 82.25 years.

Participant-flow records identify a fall during a supervised outdoor walking session in the walking group and an acute illness/hospitalization in the resistance-training group, both associated with withdrawal. The fall occurred in winter under slippery conditions. The available records do not establish the diagnosis, severity, or relationship to exercise of the acute illness/hospitalization. These events are reported separately from other reasons for withdrawal. Monthly inquiries and spontaneous reporting were used, but the available records do not support a definitive count of all adverse events or a comparison of safety across groups.

### 3.1. Baseline Characteristics

Baseline characteristics are presented in Table 1. The groups were similar in mean age and BMI, but preference-based allocation resulted in clinically relevant baseline differences that should not be interpreted using randomization-based balance assumptions.

Among participants included in the final analytic sample, the prevalence of combined pre-frailty/frailty was highest in the resistance-training group (18/20, 90.0%) compared with walking (35/50, 70.0%), Traditional Chinese exercise (12/23, 52.2%), and control (26/52, 50.0%), indicating substantial baseline imbalance and potential self-selection by functional status. The paired Traditional Chinese exercise analysis used 22 participants and therefore had a baseline prevalence of 11/22 (50.0%), as reported in the Abstract and Section 3.2. Baseline biological profiles are provided in Supplementary Table S1A,B.

### 3.2. Frailty Status and Physical Performance

**Figure 3:**
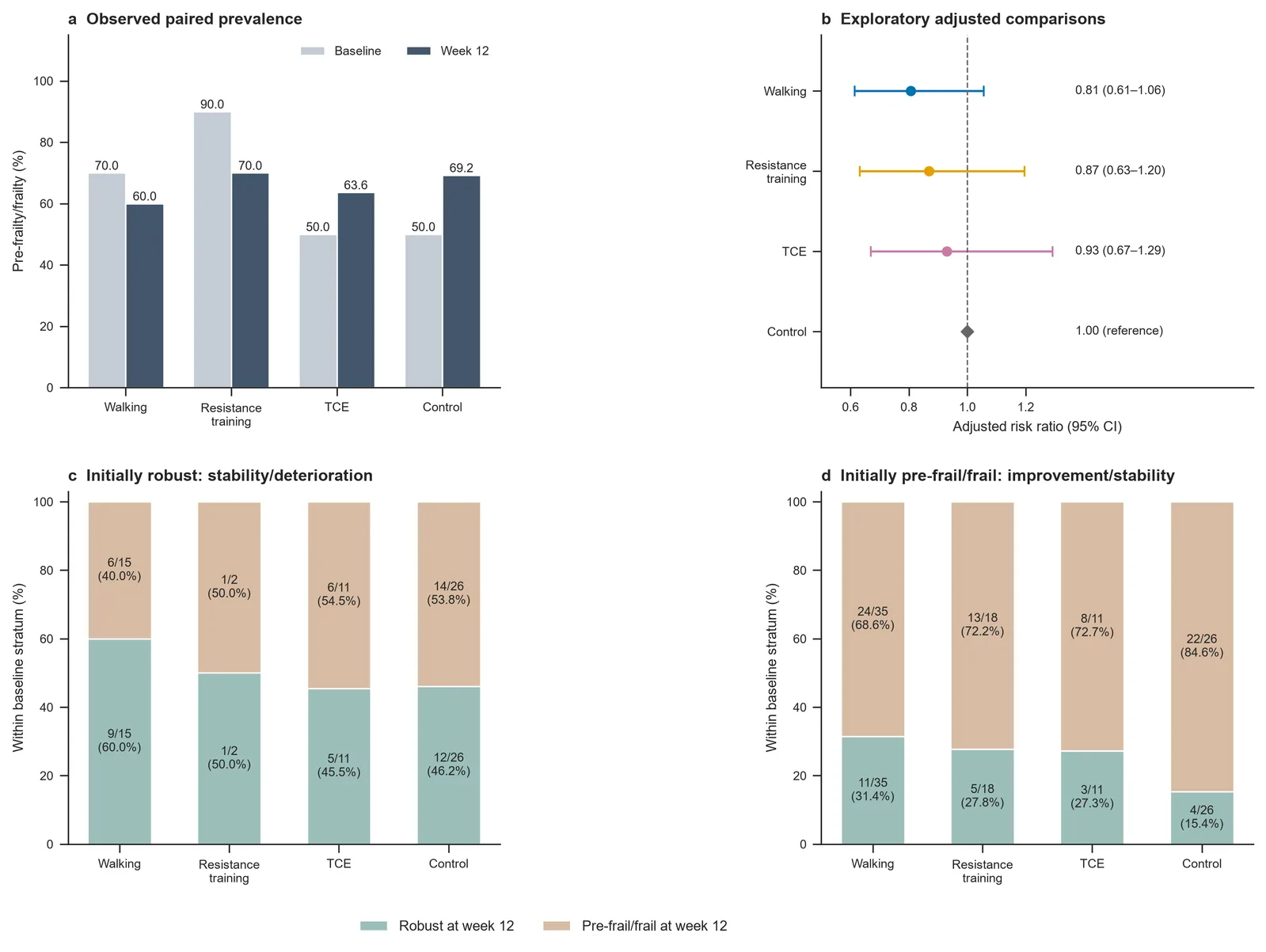
Descriptive frailty trajectories, adjusted associations, and baseline-stratified transitions. (**a**) Combined pre-frailty/frailty prevalence among paired participants (walking *n* = 50, resistance training *n* = 20, Traditional Chinese exercise *n* = 22, and control *n* = 52). (**b**) Adjusted week-12 risk ratios and 95% confidence intervals from modified Poisson models adjusted for baseline binary frailty, age, sex, and BMI. Control is fixed at RR = 1.00 as the reference and has no estimated confidence interval. (**c**) Week-12 states among initially robust participants. (**d**) Week-12 states among initially pre-frail/frail participants. Panels c and d address the unequal opportunity for binary improvement or deterioration created by baseline imbalance. Their denominators are specific to the baseline stratum; counts and percentages are shown, with Wilson intervals in Supplementary Table S8. Stability denotes unchanged binary status only; pre-frail-to-frail transitions are not resolved. Baseline differences constrain direct comparison, and adjusted frailty comparisons are inconclusive. TCE, Traditional Chinese exercise.

Paired frailty classifications were available for 50 walking, 20 resistance-training, 22 Traditional Chinese exercise, and 52 control participants. Combined pre-frailty/frailty prevalence decreased from 35/50 (70.0%) to 30/50 (60.0%) with walking (exact McNemar *p* = 0.332) and from 18/20 (90.0%) to 14/20 (70.0%) with resistance training (*p* = 0.219). It increased from 11/22 (50.0%) to 14/22 (63.6%) with Traditional Chinese exercise (*p* = 0.508) and from 26/52 (50.0%) to 36/52 (69.2%) in controls (*p* = 0.031). These within-group findings do not establish differences between groups. Adjusted week-12 comparisons remained inconclusive: walking RR 0.81 (95% CI 0.61–1.06; *p* = 0.118), resistance training RR 0.87 (95% CI 0.63–1.20; *p* = 0.389), and Traditional Chinese exercise RR 0.93 (95% CI 0.67–1.29; *p* = 0.665) (Figure 3).

Among initially pre-frail/frail participants, transitions to robust status occurred in 11/35 (31.4%) with walking, 5/18 (27.8%) with resistance training, 3/11 (27.3%) with Traditional Chinese exercise, and 4/26 (15.4%) in controls. Among initially robust participants, transitions to pre-frailty/frailty occurred in 6/15 (40.0%), 1/2 (50.0%), 6/11 (54.5%), and 14/26 (53.8%), respectively (Figure 3c,d; Supplementary Table S8). The two initially robust resistance-training participants provide very little information about deterioration. The binary outcome cannot reveal worsening within the combined pre-frail/frail category, so raw prevalence changes are not directly comparable measures of program efficacy.

Baseline physical-performance summaries for the final analytic cohort are reported in Table 1. Paired baseline and week-12 values and mean changes with 95% confidence intervals are reported in Table S2, using outcome-specific paired samples. Within-group analyses showed nominal improvements in selected outcomes, and controls also changed in several repeated performance measures. In exploratory baseline-adjusted comparisons, walking was associated with a 1.27 s lower TUG time (95% CI −2.23 to −0.31; *p* = 0.010; q = 0.039) and a 0.74 s lower 5YWT time (95% CI −1.13 to −0.36; *p* < 0.001; q = 0.001) versus the control; both remained significant after correction across eight outcomes. Resistance training was associated with 1.65 kg greater handgrip strength than the control (95% CI 0.06–3.24; nominal *p* = 0.042), but this did not remain significant after correction (q = 0.232) (Figure 4; Supplementary Table S4).

### 3.3. Hematological and Targeted Metabolomic Changes

Complete paired hematological and targeted metabolomic results are provided in Supplementary Table S3A–D. Multiple within-group molecular changes passed false-discovery-rate correction, particularly after walking and resistance training; however, several changes, including short-chain fatty acid, steroid, and amino acid measures, were also present in controls. These overlapping temporal patterns limited exercise-specific interpretation. Statistical significance alone does not demonstrate biological importance or exercise-specific adaptation.

**Figure 5:**
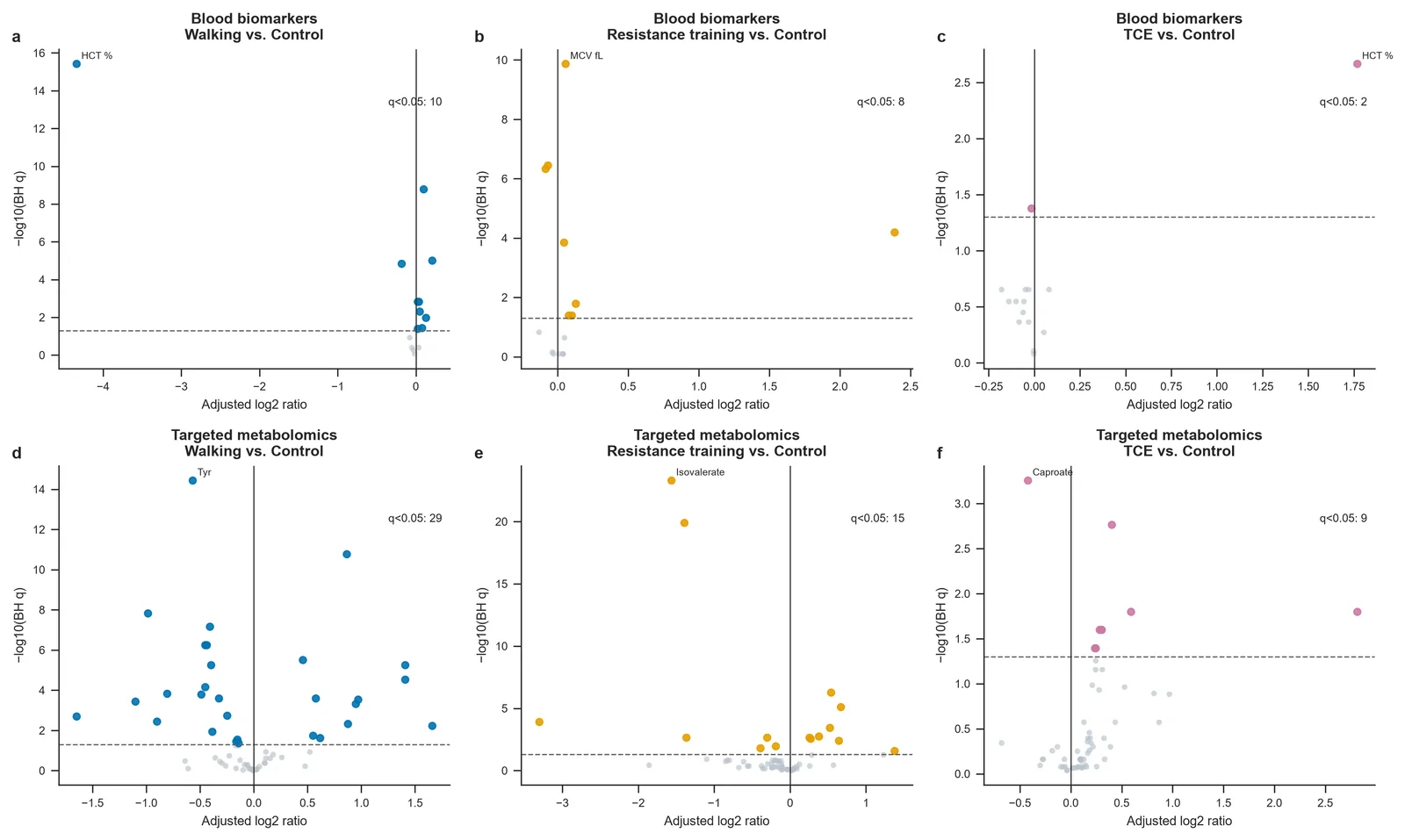
Exploratory adjusted molecular comparisons at week 12. Blood biomarker comparisons: (**a**) walking vs. control; (**b**) resistance training vs. control; (**c**) Traditional Chinese exercise (TCE) vs. control. Targeted metabolomics comparisons: (**d**) walking vs. control; (**e**) resistance training vs. control; (**f**) TCE vs. control. Volcano plots show adjusted log2 ratios and Benjamini–Hochberg q values for blood biomarkers and targeted metabolites. Differences in intervention timing and potential laboratory-batch effects cannot be separated from exercise-group differences because batch identifiers are unavailable. False discovery rate (FDR) correction does not address this confounding. These sensitivity analyses are not evidence of modality-specific biological signatures or causal effects.

Exploratory baseline-adjusted molecular comparisons produced numerous nominal and false-discovery-rate-corrected differences (Figure 5). Because the intervention groups originated from different recruitment periods and analytical-batch identifiers were not available in the analysis dataset, these contrasts could not distinguish exercise modality effects from cohort or laboratory-batch effects and were therefore treated as sensitivity analyses rather than evidence of intervention efficacy. No baseline values were available in the resistance-training group for nine measures in Table S1A: CHO, HCRP, HDLC, LDLC, TG, insulin, testosterone, cortisol, and IL-6. These measures could not contribute to baseline-adjusted comparisons for that group; differences in the available panels limit comparisons of molecular patterns across programs.

Accordingly, molecular findings describe variation over time and exploratory adjusted associations. Dietary intake, medication use, baseline health, inflammation, characteristics of participants recruited at different times, and technical variation remain alternative explanations. Their biological and clinical significance requires confirmation in studies with harmonized sampling and laboratory procedures.

### 3.4. Gut Microbiome Changes

**Figure 6:**
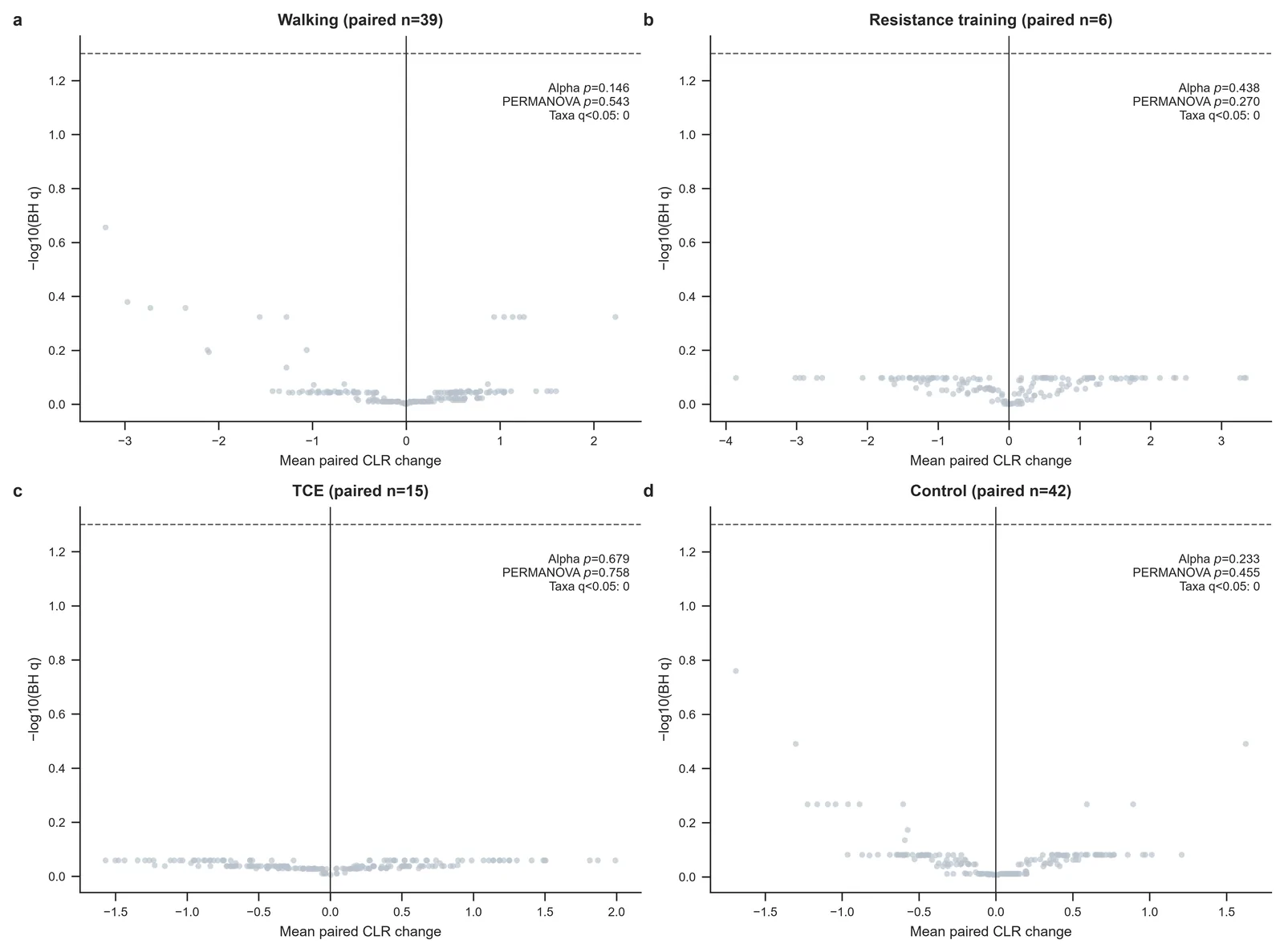
Paired gut-microbiome changes over 12 weeks: (**a**) walking (39 paired participants); (**b**) resistance training (6 paired participants); (**c**) Traditional Chinese exercise (15 paired participants); and (**d**) control (42 paired participants). Each point represents a microbial taxon, with the mean paired centered-log-ratio (CLR) change from baseline to week 12 on the horizontal axis and −log10 of the Benjamini–Hochberg-adjusted q value on the vertical axis. The vertical line indicates zero change, and the horizontal dashed line marks q = 0.05. Annotations within each panel report the P values for Shannon alpha-diversity comparisons and paired-label permutation PERMANOVA, together with the number of taxa meeting q < 0.05. Nonsignificant tests do not demonstrate equivalence or absence of change; the small and selectively available paired samples limit inference. TCE, Traditional Chinese exercise; PERMANOVA, permutational multivariate analysis of variance.

Paired microbiome data were available for 39/50 walking participants (78.0%), 6/20 resistance-training participants (30.0%), 15/23 Traditional Chinese exercise participants (65.2%), and 42/52 controls (80.8%). Shannon diversity tests were not significant (all *p* > 0.14), paired-label permutation PERMANOVA did not detect Bray–Curtis compositional shifts (all *p* ≥ 0.27), and no individual taxon survived Benjamini–Hochberg correction (Figure 6). These analyses provide insufficient evidence to determine whether microbiome changes occurred, particularly with only six resistance-training pairs.

Baseline comparisons for participants with versus without paired microbiome profiles are shown in Supplementary Table S9A. In resistance training, the paired subgroup was older on average (84.8 versus 80.8 years) and had a lower mean BMI (21.9 versus 24.0 kg/m^2^). In controls, baseline pre-frailty/frailty was present in 18/42 (42.9%) with paired profiles versus 8/10 (80.0%) without pairs. Of 43 participants without paired profiles, 36 linked only at baseline, 5 only at week 12, and 2 at neither time point (Table S9B). Missing profiles were primarily due to participants not providing stool samples.

## 4. Discussion

Over 12 weeks, the prevalence of combined pre-frailty/frailty decreased in the walking and resistance-training groups and increased in the Traditional Chinese exercise and control groups. Adjusted comparisons were imprecise, with all frailty risk-ratio confidence intervals including 1. Exercise studies in very old, hospitalized, and community-dwelling populations provide a basis for examining these trajectories [10,11,12,13,14,15]. In the present study, however, participants selected their programs and differed substantially in baseline frailty, limiting conclusions about comparative benefit.

Baseline frailty helps explain the apparent differences in prevalence trajectories. In the resistance-training group, 18 out of 20 participants were already pre-frail/frail, leaving only 2 who could cross from robust to pre-frail/frail status. By comparison, 26 out of 52 controls were initially robust. Stratified transitions showed both improvement and deterioration during follow-up. These binary categories do not capture progression from pre-frailty to frailty, which may occur despite an unchanged group classification.

Walking was associated with faster TUG and 5YWT performance than the control after adjustment for baseline performance, age, sex, and BMI and correction across eight outcomes. The handgrip-strength difference associated with resistance training did not remain significant after correction. These observations are relevant to evidence supporting aerobic, multicomponent, and resistance exercise for functional health in older adults [10,13,14,15,16], although self-selection and repeated-testing effects may have influenced the present estimates. A previous report involving 26 women aged ≥80 years described improvements in flexibility, lower-limb strength, and cardiorespiratory endurance after a 12-week brisk walking program. Its uncontrolled design limited the attribution of these changes to exercise alone [17].

Blood and metabolomic changes occurred in both exercise participants and controls. Diet, usual activity, medication use, baseline health, and technical variation may contribute to these temporal patterns. Previous studies have examined inflammatory, nutritional, and circulating biomarkers in relation to exercise and frailty [12,13,18,19,20]. In this study, differences in recruitment and intervention timing and unavailable laboratory-batch identifiers prevented the separation of program-related associations from technical and cohort variation.

SCFAs, steroid-related metabolites, vitamins, and amino acids are relevant to metabolic and nutritional health, and metabolomic profiles have been associated with frailty [21,22,23,24]. The circulating measurements reported here describe concentrations rather than pathway activity or nutrient intake. They therefore provide candidate observations for further study but cannot identify distinct mechanisms linking each exercise program to frailty.

We found no statistically significant changes in microbial diversity or individual taxa after multiplicity correction. The small paired samples, including only six participants in resistance training, limit the precision of these findings. Participants with paired profiles also differed from those without paired profiles in some baseline characteristics. Diet, medication use, age, and gastrointestinal health may further influence microbial composition. These limitations should be considered alongside the varied associations reported in the microbiome and frailty literature [25,26,27,28]. A study of adults aged ≥80 years participating in a 12-week walking program reported post-intervention microbiota differences between groups defined by nutritional status. The absence of a non-exercise control group and substantial attrition limited causal interpretation [29].

Repeated assessment across clinical, circulating molecular, and microbial measures provides information on functional trajectories and biological variation in a very old residential population. The control observations help characterize changes occurring without a structured exercise program and provide estimates for planning larger studies.

The main limitation was preference-based allocation. Functional capacity, health, motivation, and previous exercise experience may have influenced both program choice and subsequent outcomes. Models accounted for baseline outcome values, age, sex, and BMI, but residual confounding by nutrition, cognition, medications, comorbidity, and prior activity remains possible. Group-specific intervention timing may also have introduced seasonal or calendar-time differences.

All exercise programs were supervised by an on-site intervention team. However, attendance was not systematically documented, and actual received exercise dose or compliance with the planned attendance threshold could not be verified or compared across groups. Differences in received exercise dose may have contributed to the group trajectories, including the less favorable frailty pattern in Traditional Chinese exercise. Supervision alone does not establish comparable exposure. Analyses restricted to participants with week-12 assessments may introduce selection bias, particularly when withdrawal is related to illness or intervention tolerance. The shared nursing-home environment and generally stable meals, medications, and routine activities may have reduced environmental variation, but quantitative longitudinal records were unavailable. Adverse-event records were insufficient to establish complete ascertainment, severity classification, or comparative safety.

Other limitations include the single-facility setting, pragmatic recruitment without an a priori sample size calculation, and small samples for several analyses. Complete-case analyses may be biased when data availability is related to health or response. Multiple exploratory outcomes and possible learning effects warrant caution when interpreting individual associations. The molecular findings are further limited by uncertain laboratory-batch comparability. The resistance-training group lacked baseline data for nine biochemical measures in Table S1A, restricting the set of molecular outcomes that could be compared with controls.

## 5. Conclusions

Walking and resistance-training participants had declining pre-frailty/frailty prevalence over 12 weeks, whereas prevalence increased in controls. Adjusted frailty comparisons remained inconclusive. Walking was associated with better follow-up performance on two mobility tests, but the non-randomized design limits causal interpretation. Molecular and microbial findings require confirmation in larger studies with comparable sampling schedules and documented intervention exposure.

## Figures and Tables

**Table 1:** Baseline characteristics of participants by study group.

Variable	Walking (*n* = 50)	Resistance Training (*n* = 20)	Traditional Chinese Exercise (*n* = 23)	Control (*n* = 52)
Sex (male/female)	14/36	7/13	10/13	23/29
Age (years)	82.04 ± 5.81	82.00 ± 7.37	81.61 ± 5.88	82.83 ± 6.49
BMI (kg/m^2^)	23.36 ± 3.36	23.40 ± 3.64	25.16 ± 3.14 (*n* = 22)	23.94 ± 3.90 (*n* = 50)
Pre-frailty/frailty, *n* (%)	35 (70.0%)	18 (90.0%)	12 (52.2%)	26 (50.0%)
Handgrip strength (kg)	21.85 ± 6.40	21.14 ± 5.46	21.98 ± 6.86	22.37 ± 7.01
Back scratch test (cm)	−6.12 ± 12.69	−6.40 ± 14.79	−10.02 ± 10.59	−9.68 ± 13.35
Chair sit and reach (cm)	0.93 ± 11.70	0.41 ± 14.07	−2.55 ± 9.11	−2.50 ± 11.97 (*n* = 51)
Timed Up and Go (s)	10.67 ± 3.63	10.89 ± 5.03	11.93 ± 4.88 (*n* = 21)	10.80 ± 2.42
6MWT (m)	414.91 ± 116.40 (*n* = 49)	430.60 ± 147.73	383.68 ± 109.93 (*n* = 22)	386.16 ± 78.15 (*n* = 51)
5YWT (s)	4.25 ± 1.13	4.35 ± 1.43	4.35 ± 1.28	4.14 ± 0.79
SPPB	9.30 ± 2.31	8.95 ± 2.72	9.35 ± 2.62	9.87 ± 1.87
Berg Balance Scale score (points)	52.54 ± 2.94	51.90 ± 3.55	50.96 ± 8.00	52.31 ± 4.60

Values are the mean ± SD or *n* (%). Table 1 summarizes available baseline measurements in the final analytic cohort. Cell-specific *n* is shown when it differs from the group total. Table S2 includes only participants with both baseline and week-12 measurements for the relevant outcome; its baseline values may therefore differ when the paired subset is smaller.

## Data Availability

The data supporting the findings of this study are available from the corresponding author upon reasonable request. Participant-level data are not publicly available because they contain potentially identifying health information and public sharing was not included in the consent obtained.

## Supplementary Materials

The following supporting information can be downloaded at: https://www.mdpi.com/article/10.3390/nu18183052/s1, Tables S1A,B. Baseline biological characteristics; Table S2. Paired physical-performance changes with 95% confidence intervals; Tables S3A–D. Paired molecular changes; Table S4. Adjusted physical-performance comparisons; Table S5. Binary frailty transitions and adjusted risk ratios; Tables S6A,B. Exploratory adjusted molecular comparisons; Tables S7A,B. Microbiome results; Table S8. Baseline-stratified frailty transitions; Tables S9A,B. Paired microbiome availability and baseline characteristics; Table S10. Availability of selected baseline covariate fields.
