# Supplementary material for: Frailty, Physical Performance, and Molecular Changes in Older Adults Participating in Different Exercise Programs: A 12-Week Preference-Based Non-Randomized Study
Source: Nutrients. 2026 Sep 18;18(18):3052. doi: 10.3390/nu18183052 (PMC13610386; doi:10.3390/nu18183052)
Supplement: Supplementary file 1 [file nutrients-18-03052-s001.zip › nutrients-4532394-supplementary.pdf]

Supplementary Material

Frailty, Physical Performance, and Molecular Changes in Older Adults Participating in Different Exercise Programs: A 12-Week Preference-Based Non-Randomized Study

Analytical population: walking n=50, resistance training n=20, Traditional Chinese exercise n=23, and control n=52. Outcome-specific paired sample sizes are reported in each table.

Supplementary statistical notes

Frailty is the primary outcome. Paired analyses are descriptive; physical-performance and molecular outcomes and all adjusted between-group comparisons are exploratory. Baseline-stratified transitions and missingness summaries were conducted post hoc. Family-specific Benjamini–Hochberg correction does not provide study-wide error control or address confounding. Paired mean-change confidence intervals are descriptive, not multiplicity-adjusted.

Table S1A. Baseline hematological characteristics by study group

Variable	Walking	Resistance training	Traditional Chinese exercise	Control
CHO	4.63 ± 1.13	Not available	3.83 ± 0.98	3.99 ± 0.91
HCRP	0.25 ± 0.83	Not available	Not available	0.03 ± 0.08
HDLC	1.48 ± 0.36	Not available	1.45 ± 0.36	1.59 ± 0.36
LDLC	2.81 ± 1.03	Not available	2.40 ± 0.85	2.33 ± 0.78
TG	1.33 ± 0.68	Not available	1.18 ± 0.60	1.17 ± 0.65
Insulin	65.21 ± 62.73	Not available	6.51 ± 1.52	24.42 ± 32.89
Testo(ng/mL)	4.32 ± 5.73	Not available	Not available	10.19 ± 8.10
Cortisol(ug/dL)	481.17 ± 159.91	Not available	Not available	472.21 ± 82.33
IL-6	6.92 ± 26.22	Not available	Not available	4.17 ± 3.52
WBC 10^9/L	6.40 ± 1.78	6.47 ± 2.02	5.87 ± 1.23	6.20 ± 1.56
Neu# 10^9/L	3.53 ± 1.26	3.55 ± 1.63	3.20 ± 0.77	3.38 ± 1.12
Lym# 10^9/L	2.25 ± 0.62	2.33 ± 0.65	2.13 ± 0.68	2.21 ± 0.66
Mon# 10^9/L	0.39 ± 0.14	0.42 ± 0.15	0.33 ± 0.08	0.39 ± 0.12
Eos# 10^9/L	0.20 ± 0.17	0.16 ± 0.13	0.19 ± 0.20	0.20 ± 0.11
Bas# 10^9/L	0.02 ± 0.01	0.02 ± 0.01	0.02 ± 0.00756	0.02 ± 0.01
Neu% %	54.45 ± 8.12	53.34 ± 9.47	54.76 ± 7.72	53.99 ± 7.95
Lym% %	36.04 ± 8.55	37.50 ± 8.84	36.15 ± 7.81	36.12 ± 7.71
Mon% %	6.12 ± 1.50	6.44 ± 1.27	5.68 ± 1.14	6.33 ± 1.50
Eos% %	3.01 ± 1.97	2.45 ± 1.71	3.05 ± 2.72	3.19 ± 1.51
Bas% %	0.38 ± 0.21	0.26 ± 0.12	0.36 ± 0.15	0.37 ± 0.22
RBC 10^12/L	3.73 ± 0.61	3.62 ± 0.72	4.36 ± 0.35	4.17 ± 0.69
HGB g/L	115.86 ± 18.96	113.90 ± 19.81	133.67 ± 9.19	128.12 ± 18.43
HCT %	34.75 ± 5.72	34.12 ± 6.39	42.79 ± 2.59	39.96 ± 6.55
MCV fL	93.15 ± 3.82	94.47 ± 3.83	98.31 ± 4.70	96.02 ± 4.50
MCH pg	31.10 ± 1.34	31.65 ± 1.48	30.71 ± 1.65	30.91 ± 1.69
MCHC g/L	333.98 ± 8.92	334.95 ± 8.48	312.40 ± 6.99	322.06 ± 13.31
RDW-CV %	12.72 ± 0.64	12.45 ± 0.55	13.60 ± 0.92	12.99 ± 0.82
RDW-SD fL	42.64 ± 2.28	42.42 ± 2.26	49.59 ± 4.85	45.67 ± 4.00
PLT 10^9/L	228.72 ± 67.74	249.55 ± 70.60	200.60 ± 73.38	226.62 ± 52.37
MPV fL	10.19 ± 1.02	9.95 ± 1.09	10.39 ± 1.17	9.62 ± 1.01
PDW	16.12 ± 0.36	16.08 ± 0.37	16.22 ± 0.43	16.04 ± 0.35
PCT %	0.23 ± 0.06	0.24 ± 0.06	0.20 ± 0.06	0.22 ± 0.05

Variable	Walking	Resistance training	Traditional Chinese exercise	Control
P-LCC 10^9/L	61.32 ± 15.17	63.05 ± 18.24	56.07 ± 17.27	52.87 ± 15.91
P-LCR %	28.14 ± 7.61	26.32 ± 7.89	29.55 ± 8.82	24.12 ± 7.42

Values are mean ± SD from available baseline observations. No baseline significance tests are shown because allocation was non-randomized and preference based. "Not available" indicates that no baseline value was available for that group and variable. No baseline values were available in the resistance-training group for nine measures in Table S1A: CHO, HCRP, HDLC, LDLC, TG, insulin, testosterone, cortisol, and IL-6. These measures could not contribute to baseline-adjusted comparisons for that group; differences in available panels limit comparisons of molecular patterns across programs. These baseline summaries were regenerated from the same final analysis dataset used for Table 1 and the paired and adjusted analyses. NA denotes an undefined SD when only one observation is available.

Table S1B. Baseline targeted metabolomic characteristics by study group

Variable	Walking	Resistance training	Traditional Chinese exercise	Control
tHCY	12.67 ± 6.25	12.49 ± 8.48	11.05 ± 2.81	12.48 ± 6.17
Acetate	8693.20 ± 6410.96	11218.41 ± 8308.49	9369.67 ± 2373.73	11538.74 ± 7072.65
Propionate	294.71 ± 208.11	260.21 ± 189.66	462.95 ± 61.35	421.07 ± 310.91
Isobutyrate	170.99 ± 82.40	151.53 ± 47.15	180.81 ± 17.68	178.40 ± 58.45
butyrate	423.09 ± 198.43	374.81 ± 110.09	460.93 ± 66.63	456.34 ± 155.21
Isovalerate	170.55 ± 111.25	233.44 ± 179.90	197.41 ± 25.84	186.67 ± 84.78
Valerate	83.73 ± 56.59	115.47 ± 88.07	105.46 ± 18.96	103.11 ± 49.62
Caproate	369.69 ± 152.53	376.09 ± 94.10	384.65 ± 57.32	400.41 ± 135.24
17α-Hydroxyprogesterone	0.90 ± 0.51	0.92 ± 0.52	0.58 ± 0.63	0.83 ± 0.57
Androstenedione	0.82 ± 0.33	0.80 ± 0.31	0.47 ± 0.24	0.66 ± 0.27
Aldosterone	0.12 ± 0.09	0.14 ± 0.09	213.88 ± 58.57	115.71 ± 110.76
Cortisone	20.17 ± 5.67	20.01 ± 6.15	129.35 ± 29.91	77.76 ± 58.33
Corticosterone	8.23 ± 5.07	9.46 ± 6.23	14.51 ± 2.66	11.94 ± 6.02
Dehydroepiandrosterone	2.48 ± 1.70	2.39 ± 1.53	1.55 ± 0.79	1.60 ± 0.94
11-Deoxycorticosterone	0.07 ± 0.05	0.08 ± 0.07	42.78 ± 46.12	28.81 ± 42.06
Estrone	0.03 ± 0.02	0.03 ± 0.02	19.51 ± 13.22	11.62 ± 12.74
Estradiol	0.01 ± 0.01	0.01 ± 0.01	3.96 ± 11.02	7.79 ± 16.88
Cortisol	195.71 ± 59.76	205.14 ± 73.81	3.89 ± 1.95	85.81 ± 97.82
Progesterone	0.07 ± 0.08	0.09 ± 0.10	0.04 ± 0.07	0.06 ± 0.06
11-Deoxycortisol	0.81 ± 0.54	0.94 ± 0.69	0.53 ± 0.33	0.64 ± 0.41
Testosterone	1.40 ± 2.27	1.46 ± 1.78	596.77 ± 1290.79	1090.41 ± 1927.83
VA	531.10 ± 148.92	533.96 ± 144.68	588.57 ± 103.06	537.13 ± 119.69
VD2	1.29 ± 1.73	1.75 ± 2.51	0.65 ± 0.49	1.27 ± 1.65
VD3	19.89 ± 7.87	24.04 ± 9.55	20.14 ± 9.03	22.36 ± 12.37
VE	11.60 ± 3.60	11.78 ± 3.93	16.86 ± 3.39	14.37 ± 5.13
VK	0.96 ± 0.56	0.98 ± 0.55	2.91 ± 2.14	1.66 ± 1.06
5-MTHF	11.12 ± 11.63	13.10 ± 12.18	25.60 ± 16.77	17.42 ± 17.15
VB1	1.77 ± 3.02	2.07 ± 2.17	1.98 ± 2.36	2.25 ± 3.44
VB2	29.36 ± 38.66	25.23 ± 34.93	36.25 ± 49.41	23.70 ± 29.83
VB5	119.32 ± 85.84	146.58 ± 125.19	128.23 ± 133.39	108.76 ± 54.72
VB6PA	7.01 ± 9.34	8.11 ± 12.10	11.24 ± 17.45	8.45 ± 8.65
Ala	437.19 ± 131.24	423.96 ± 121.41	409.69 ± 154.22	370.20 ± 124.77
Arg	80.18 ± 25.33	79.12 ± 21.80	45.45 ± 14.00	64.92 ± 26.51
Asn	34.73 ± 8.65	34.06 ± 6.69	48.56 ± 7.60	43.95 ± 10.89
Asp	6.04 ± 3.63	5.30 ± 3.45	3.83 ± 1.44	4.64 ± 3.36
Cit	36.34 ± 14.54	31.87 ± 16.44	35.42 ± 12.02	37.75 ± 13.65
Gln	374.01 ± 71.39	384.71 ± 71.69	518.64 ± 70.45	446.05 ± 119.39
Glu	57.57 ± 35.16	54.27 ± 37.35	40.19 ± 14.14	47.45 ± 32.78

Variable	Walking	Resistance training	Traditional Chinese exercise	Control
His	57.96 ± 13.23	61.30 ± 11.19	82.28 ± 11.41	70.54 ± 16.66
Ile	75.69 ± 22.79	73.01 ± 19.72	84.00 ± 20.17	75.61 ± 18.32
Leu	119.47 ± 31.90	115.39 ± 26.35	88.15 ± 12.50	97.32 ± 29.20
Lys	201.06 ± 56.57	199.45 ± 41.67	239.45 ± 36.35	215.47 ± 37.56
Met	24.33 ± 4.79	23.12 ± 4.14	21.81 ± 4.61	23.14 ± 4.48
Orn	47.15 ± 29.20	37.72 ± 12.93	76.01 ± 34.01	66.43 ± 36.55
Phe	64.00 ± 13.29	64.65 ± 11.55	36.96 ± 6.91	49.34 ± 17.02
Pro	253.07 ± 147.58	244.05 ± 114.58	211.55 ± 59.72	234.08 ± 109.93
Ser	70.08 ± 29.97	65.12 ± 22.99	63.73 ± 15.15	70.15 ± 30.39
Thr	104.58 ± 25.63	104.56 ± 19.61	105.52 ± 32.74	108.65 ± 40.68
Trp	43.53 ± 9.47	40.64 ± 7.78	43.19 ± 5.50	42.39 ± 7.14
Tyr	69.51 ± 12.37	66.27 ± 13.19	63.25 ± 10.31	62.79 ± 13.52
Val	209.80 ± 50.47	211.97 ± 47.89	270.00 ± 38.55	221.34 ± 41.03
66 Zn [ppb]	1014.78 ± 832.72	1172.01 ± 1353.76	1422.63 ± 220.10	1124.30 ± 332.29
55 Mn [ppb]	92.16 ± 205.04	153.38 ± 264.63	1.22 ± 0.25	4.79 ± 19.86
24 Mg [ppm]	24.03 ± 6.22	24.47 ± 9.43	19798.30 ± 1453.62	11672.27 ± 10309.64
63 Cu [ppb]	1031.62 ± 240.27	1076.07 ± 274.25	910.17 ± 90.26	968.83 ± 202.49
56 Fe [ppb]	1258.71 ± 449.32	1306.17 ± 561.45	1065.46 ± 277.93	1125.07 ± 374.76
78 Se [ppb]	103.73 ± 27.87	101.66 ± 21.71	126.72 ± 26.37	111.11 ± 21.75
52 Cr [ppb]	1.78 ± 7.66	3.66 ± 12.45	0.93 ± 0.22	1.42 ± 4.09
59 Co [ppb]	0.28 ± 0.47	0.41 ± 0.65	0.18 ± 0.10	0.18 ± 0.10
88 Sr [ppb]	96.76 ± 391.73	185.93 ± 640.52	43.38 ± 9.82	45.22 ± 14.98
127 I [ppb]	49.76 ± 10.46	47.57 ± 9.89	56.94 ± 13.23	48.84 ± 10.10
44 Ca [ppm]	30.24 ± 103.59	54.82 ± 169.22	15131.89 ± 672.57	8368.64 ± 7365.70
208 Pb [ppb]	6.74 ± 15.03	14.74 ± 24.69	2.08 ± 0.48	1.85 ± 0.77
201 Hg [ppb]	0.16 ± 0.10	0.18 ± 0.09	0.10 ± 0.04	0.12 ± 0.07
111 Cd [ppb]	0.31 ± 0.81	0.68 ± 1.45	0.02 ± 0.02	0.13 ± 0.10
75 As [ppb]	1.49 ± 2.56	1.24 ± 1.52	1.39 ± 1.37	1.30 ± 1.49
205 Tl [ppb]	Not available	Not available	0.00786 ± 0.00886	0.06 ± 0.05
51 V [ppb]	0.62 ± 3.06	1.51 ± 4.89	0.49 ± 0.08	0.36 ± 0.24
71 Ga [ppb]	1.34 ± NA	1.34 ± NA	0.13 ± 0.05	0.13 ± 0.07

Values are mean ± SD from available baseline observations. No baseline significance tests are shown because allocation was non-randomized and preference based. "Not available" indicates that no baseline value was available for that group and variable. These baseline summaries were regenerated from the same final analysis dataset used for Table 1 and the paired and adjusted analyses. NA denotes an undefined SD when only one observation is available.

Table S2. Physical-performance outcomes: paired within-group changes

Outcome	Group	Paired n	Baseline, mean ± SD	Week 12, mean ± SD	Mean change (95% CI)	Paired t P	Wilcoxon P
Handgrip strength (kg)	Control	52	22.37 ± 7.01	23.01 ± 7.25	0.64 (-0.48 to 1.77)	0.258	0.056
Handgrip strength (kg)	Resistance training	20	21.14 ± 5.46	23.61 ± 5.62	2.48 (1.22 to 3.73)	<0.001	<0.001
Handgrip strength (kg)	Traditional Chinese exercise	22	22.05 ± 7.01	20.31 ± 8.48	-1.73 (-4.52 to 1.06)	0.211	0.603
Handgrip strength (kg)	Walking	50	21.85 ± 6.40	22.64 ± 7.01	0.79 (0.03 to 1.54)	0.041	0.070
Back scratch (cm)	Control	47	-8.14 ± 12.32	-7.84 ± 13.48	0.30 (-1.10 to 1.70)	0.669	0.559
Back scratch (cm)	Resistance training	20	-6.40 ± 14.79	-7.17 ± 14.16	-0.78 (-4.15 to 2.60)	0.636	0.588
Back scratch (cm)	Traditional Chinese exercise	15	-10.27 ± 12.24	-10.29 ± 10.44	-0.02 (-2.13 to 2.09)	0.984	0.754
Back scratch (cm)	Walking	49	-6.24 ± 12.79	-4.18 ± 13.72	2.06 (-0.73 to 4.84)	0.144	0.278
Chair sit-and-reach (cm)	Control	47	-1.94 ± 12.19	-0.95 ± 11.09	0.99 (-1.61 to 3.59)	0.446	0.340

Outcome	Group	Paired n	Baseline, mean ± SD	Week 12, mean ± SD	Mean change (95% CI)	Paired t P	Wilcoxon P
Chair sit-and-reach (cm)	Resistance training	20	0.41 ± 14.07	4.12 ± 14.50	3.71 (0.92 to 6.50)	0.012	0.017
Chair sit-and-reach (cm)	Traditional Chinese exercise	20	-1.69 ± 9.05	-1.62 ± 11.38	0.06 (-2.94 to 3.06)	0.967	0.984
Chair sit-and-reach (cm)	Walking	50	0.93 ± 11.70	3.63 ± 12.07	2.70 (-0.02 to 5.41)	0.052	0.058
TUG (s)	Control	52	10.80 ± 2.42	11.37 ± 3.44	0.57 (-0.16 to 1.31)	0.123	0.201
TUG (s)	Resistance training	20	10.89 ± 5.03	10.20 ± 4.02	-0.69 (-1.98 to 0.60)	0.277	0.177
TUG (s)	Traditional Chinese exercise	20	11.82 ± 4.99	12.49 ± 4.99	0.66 (-0.13 to 1.45)	0.096	0.073
TUG (s)	Walking	50	10.67 ± 3.63	9.90 ± 2.70	-0.77 (-1.46 to -0.07)	0.032	0.004
6MWT (m)	Control	49	389.36 ± 75.48	405.34 ± 79.79	15.98 (1.45 to 30.50)	0.032	0.030
6MWT (m)	Resistance training	19	437.58 ± 148.35	421.68 ± 127.31	-15.89 (-84.38 to 52.59)	0.632	0.732
6MWT (m)	Traditional Chinese exercise	21	390.52 ± 107.74	405.24 ± 118.75	14.71 (-15.15 to 44.58)	0.316	0.539
6MWT (m)	Walking	48	417.37 ± 116.34	430.04 ± 97.92	12.68 (-17.44 to 42.79)	0.401	0.056
5YWT (s)	Control	52	4.14 ± 0.79	4.29 ± 1.31	0.14 (-0.13 to 0.42)	0.291	0.579
5YWT (s)	Resistance training	20	4.35 ± 1.43	4.12 ± 1.63	-0.22 (-0.72 to 0.27)	0.355	0.202
5YWT (s)	Traditional Chinese exercise	22	4.30 ± 1.29	4.87 ± 2.07	0.57 (0.03 to 1.10)	0.038	0.054
5YWT (s)	Walking	50	4.25 ± 1.13	3.58 ± 0.98	-0.67 (-0.92 to -0.43)	<0.001	<0.001
SPPB (points)	Control	48	9.88 ± 1.94	9.92 ± 2.01	0.04 (-0.46 to 0.54)	0.867	0.877
SPPB (points)	Resistance training	20	8.95 ± 2.72	9.45 ± 2.44	0.50 (-0.31 to 1.31)	0.212	0.221
SPPB (points)	Traditional Chinese exercise	19	9.37 ± 2.81	9.84 ± 2.32	0.47 (-0.32 to 1.27)	0.226	0.296
SPPB (points)	Walking	50	9.30 ± 2.31	10.18 ± 2.09	0.88 (0.39 to 1.37)	<0.001	<0.001
Berg balance (points)	Control	51	52.27 ± 4.64	53.69 ± 2.14	1.41 (0.37 to 2.46)	0.009	0.011
Berg balance (points)	Resistance training	20	51.90 ± 3.55	53.05 ± 3.58	1.15 (0.27 to 2.03)	0.013	0.014
Berg balance (points)	Traditional Chinese exercise	21	50.76 ± 8.31	53.24 ± 3.39	2.48 (-1.23 to 6.18)	0.178	0.125
Berg balance (points)	Walking	49	52.51 ± 2.96	53.04 ± 3.37	0.53 (-0.36 to 1.42)	0.236	0.064

Change is week 12 minus baseline, with two-sided 95% t-based confidence intervals computed from the individual paired differences. Negative TUG and 5YWT changes indicate faster performance; positive values indicate higher scores/distances or strength for the other tests. Paired t and Wilcoxon P values are unadjusted descriptive results. Confidence intervals are not multiplicity-adjusted. Table S2 was regenerated from the same final analysis dataset as Table 1. Baseline values agree when the participant sets are identical; differences for smaller paired subsets reflect missing follow-up measurements.

Tables S3A–S3D. Paired hematological and targeted metabolomic changes

All observed positive concentrations were log2 transformed. P values test paired log-ratios; q values are Benjamini–Hochberg adjusted within the molecular family and study group. These are descriptive within-group analyses and do not establish an exercise-specific effect.

Table S3A. Walking: paired molecular changes

Analyte	Paired n	Baseline mean	Week-12 mean	log2 fold change	Fold change	P	q
tHCY	48	12.300	13.232	0.060	1.043	0.300	0.457
Acetate	48	8664.6	4599.5	-0.390	0.763	0.139	0.259
Propionate	47	296.5	468.6	1.011	2.015	<0.001	<0.001
Isobutyrate	48	170.4	152.0	-0.063	0.958	0.453	0.604
butyrate	48	421.1	384.9	-0.035	0.976	0.653	0.760
Isovalerate	45	174.6	229.4	0.724	1.652	<0.001	<0.001
Valerate	45	85.640	167.2	1.375	2.593	<0.001	<0.001
Caproate	48	358.0	807.2	1.262	2.399	<0.001	<0.001
17α-Hydroxyprogesterone	48	0.897	0.829	-0.405	0.755	0.081	0.163

Analyte	Paired n	Baseline mean	Week-12 mean	log2 fold change	Fold change	P	q
Androstenedione	48	0.808	0.742	-0.182	0.882	0.163	0.282
Aldosterone	48	0.121	0.079	-0.722	0.606	0.002	0.004
Cortisone	48	20.266	19.422	-0.047	0.968	0.623	0.752
Corticosterone	48	8.042	7.621	-0.163	0.893	0.477	0.623
Dehydroepiandrosterone	48	2.369	2.570	0.085	1.061	0.673	0.769
11-Deoxycorticosterone	48	0.064	0.057	-0.185	0.880	0.347	0.517
Estrone	48	0.030	0.027	-0.118	0.921	0.573	0.705
Estradiol	48	0.011	0.030	2.348	5.090	<0.001	<0.001
Cortisol	48	193.5	113.4	-0.790	0.578	<0.001	<0.001
Progesterone	48	0.069	0.101	1.141	2.206	<0.001	0.002
11-Deoxycortisol	48	0.809	0.604	-0.418	0.748	0.022	0.049
Testosterone	48	1.446	1.751	0.091	1.065	0.861	0.895
VA	48	532.0	574.4	0.105	1.076	0.208	0.342
VD2	48	1.318	1.446	0.072	1.051	0.774	0.847
VD3	48	19.902	18.447	-0.185	0.880	0.142	0.259
VE	48	11.436	9.988	-0.196	0.873	0.032	0.069
VK	48	0.973	0.854	-0.234	0.850	0.255	0.408
5-MTHF	48	11.410	44.318	2.165	4.483	<0.001	<0.001
VB1	48	1.838	4.496	1.669	3.181	<0.001	<0.001
VB2	48	29.574	47.805	0.530	1.444	0.040	0.083
VB5	48	121.6	161.9	0.451	1.367	<0.001	0.002
VB6PA	48	7.165	17.600	1.211	2.315	<0.001	<0.001
Ala	48	438.7	427.3	-0.030	0.980	0.685	0.769
Arg	48	80.402	54.492	-0.545	0.686	<0.001	<0.001
Asn	48	34.681	41.940	0.281	1.215	<0.001	0.002
Asp	48	6.070	2.878	-1.117	0.461	<0.001	<0.001
Cit	48	35.863	31.219	-0.125	0.917	0.384	0.545
Gln	48	375.3	532.1	0.504	1.418	<0.001	<0.001
Glu	48	58.507	57.378	0.092	1.066	0.525	0.659
His	48	57.559	49.577	-0.215	0.861	0.003	0.007
Ile	48	76.228	53.119	-0.508	0.703	<0.001	<0.001
Leu	48	120.5	71.313	-0.750	0.595	<0.001	<0.001
Lys	48	201.8	158.5	-0.330	0.795	<0.001	<0.001
Met	48	24.450	20.901	-0.240	0.847	<0.001	<0.001
Orn	48	47.456	56.213	0.293	1.225	0.006	0.014
Phe	48	64.313	46.819	-0.445	0.734	<0.001	<0.001
Pro	48	257.0	193.3	-0.366	0.776	0.010	0.024
Ser	48	70.622	69.385	0.048	1.034	0.652	0.760
Thr	48	105.5	80.242	-0.401	0.757	<0.001	<0.001
Trp	48	43.898	37.164	-0.247	0.843	<0.001	0.001
Tyr	48	69.963	40.674	-0.787	0.580	<0.001	<0.001
Val	48	211.1	166.0	-0.336	0.792	<0.001	<0.001
66 Zn [ppb]	48	1014.8	872.3	-0.105	0.930	0.175	0.295
24 Mg [ppm]	48	24.029	24.016	0.009	1.006	0.867	0.895
63 Cu [ppb]	48	1031.6	1102.0	0.085	1.060	0.261	0.408
56 Fe [ppb]	48	1258.7	1271.8	0.005	1.003	0.964	0.964
78 Se [ppb]	48	103.7	107.2	0.026	1.018	0.793	0.847
52 Cr [ppb]	46	1.845	0.946	0.218	1.163	0.523	0.659
88 Sr [ppb]	48	96.761	43.817	0.018	1.012	0.921	0.935
127 I [ppb]	48	49.761	54.834	0.080	1.057	0.399	0.555

Analyte	Paired n	Baseline mean	Week-12 mean	log2 fold change	Fold change	P	q
44 Ca [ppm]	48	30.244	15.554	-0.098	0.934	0.449	0.604
201 Hg [ppb]	7	0.221	0.145	-1.044	0.485	0.121	0.236
111 Cd [ppb]	15	0.393	0.161	0.150	1.109	0.794	0.847
75 As [ppb]	15	0.514	1.017	0.745	1.676	0.160	0.282
51 V [ppb]	21	0.820	0.048	-0.444	0.735	0.365	0.531

Table S3B. Resistance training: paired molecular changes

Analyte	Paired n	Baseline mean	Week-12 mean	log2 fold change	Fold change	P	q
tHCY	18	12.230	14.645	0.310	1.240	0.001	0.004
Acetate	18	11799.7	15930.7	0.647	1.566	0.009	0.025
Propionate	18	254.7	563.0	1.526	2.880	<0.001	<0.001
Isobutyrate	18	150.7	143.3	-0.011	0.992	0.921	0.922
butyrate	18	373.1	377.7	0.069	1.049	0.557	0.678
Isovalerate	17	237.4	71.838	-1.451	0.366	<0.001	0.001
Valerate	17	117.0	35.129	-1.429	0.371	<0.001	0.001
Caproate	18	381.7	746.2	0.996	1.994	<0.001	<0.001
17α-Hydroxyprogesterone	18	0.877	0.468	-1.246	0.422	0.025	0.055
Androstenedione	18	0.741	0.492	-0.930	0.525	0.025	0.055
Aldosterone	18	0.144	0.132	-0.203	0.869	0.509	0.637
Cortisone	18	20.504	121.2	2.288	4.885	<0.001	<0.001
Corticosterone	18	9.923	16.815	0.511	1.425	0.427	0.569
Dehydroepiandrosterone	18	2.156	0.741	-1.818	0.284	<0.001	<0.001
11-Deoxycorticosterone	18	0.081	0.040	-0.859	0.551	0.011	0.029
Estrone	18	0.027	0.026	0.052	1.037	0.770	0.864
Estradiol	17	0.011	0.005	-0.356	0.781	0.565	0.678
Cortisol	18	210.3	5.636	-5.831	0.018	<0.001	<0.001
Progesterone	18	0.089	0.038	-0.307	0.808	0.577	0.679
11-Deoxycortisol	18	0.863	0.288	-1.848	0.278	<0.001	0.001
Testosterone	18	1.170	0.774	-1.010	0.496	0.043	0.084
VA	18	538.7	814.7	0.604	1.520	<0.001	<0.001
VD2	18	1.233	1.071	-0.062	0.958	0.805	0.878
VD3	18	24.956	34.088	0.475	1.390	<0.001	<0.001
VE	18	12.175	14.717	0.242	1.183	0.040	0.083
VK	18	1.024	1.298	0.305	1.235	0.024	0.055
5-MTHF	18	12.114	11.363	-0.046	0.969	0.854	0.896
VB1	18	1.917	2.887	0.504	1.418	0.243	0.374
VB2	18	21.572	16.013	-0.391	0.762	0.042	0.083
VB5	18	150.3	99.380	-0.579	0.670	<0.001	<0.001
VB6PA	18	8.505	8.874	0.205	1.153	0.453	0.591
Ala	18	417.9	377.2	-0.148	0.903	0.111	0.180
Arg	18	78.138	54.159	-0.513	0.701	<0.001	<0.001
Asn	18	33.320	39.200	0.241	1.182	0.001	0.004
Asp	18	4.791	2.632	-1.143	0.453	0.012	0.029
Cit	18	28.923	33.398	0.353	1.277	0.088	0.151
Gln	18	379.6	481.9	0.363	1.287	<0.001	<0.001
Glu	18	52.782	80.808	0.821	1.767	0.001	0.004
His	18	60.925	63.231	0.062	1.044	0.293	0.440
Ile	18	70.422	61.529	-0.205	0.868	0.004	0.012
Leu	18	112.2	70.662	-0.674	0.627	<0.001	<0.001

Analyte	Paired n	Baseline mean	Week-12 mean	log2 fold change	Fold change	P	q
Lys	18	199.8	178.4	-0.162	0.894	0.022	0.053
Met	18	22.760	23.734	0.060	1.042	0.308	0.450
Orn	18	38.356	70.521	0.897	1.862	<0.001	<0.001
Phe	18	63.431	57.094	-0.151	0.901	0.046	0.084
Pro	18	241.6	156.7	-0.621	0.650	<0.001	<0.001
Ser	18	66.853	72.532	0.175	1.129	0.165	0.260
Thr	18	106.3	109.6	0.014	1.010	0.866	0.896
Trp	18	39.529	42.270	0.072	1.051	0.374	0.522
Tyr	18	64.938	65.888	0.020	1.014	0.777	0.864
Val	18	210.0	178.6	-0.225	0.855	<0.001	0.001
66 Zn [ppb]	16	1223.6	962.5	-0.182	0.881	0.486	0.620
24 Mg [ppm]	16	24.981	20.986	-0.189	0.877	0.055	0.097
63 Cu [ppb]	16	1075.7	1058.9	0.008	1.005	0.922	0.922
56 Fe [ppb]	16	1253.7	1568.3	0.128	1.092	0.646	0.746
78 Se [ppb]	16	103.2	106.4	0.061	1.043	0.424	0.569
52 Cr [ppb]	16	4.016	8.648	1.574	2.976	0.045	0.084
88 Sr [ppb]	16	202.0	40.732	-0.083	0.944	0.854	0.896
127 I [ppb]	16	47.345	43.171	-0.119	0.921	0.109	0.180
44 Ca [ppm]	16	59.799	14.624	-0.369	0.774	0.320	0.458

Table S3C. Traditional Chinese exercise: paired molecular changes

Analyte	Paired n	Baseline mean	Week-12 mean	log2 fold change	Fold change	P	q
tHCY	11	11.316	10.990	-0.104	0.931	0.494	0.707
Acetate	11	9346.5	13457.6	0.456	1.372	0.019	0.251
Propionate	11	465.9	581.8	0.286	1.219	0.054	0.338
Isobutyrate	11	181.4	201.7	0.139	1.101	0.121	0.338
butyrate	11	457.2	532.8	0.203	1.151	0.086	0.338
Isovalerate	11	198.1	255.8	0.362	1.285	0.001	0.085
Valerate	11	107.4	99.609	-0.177	0.885	0.363	0.667
Caproate	11	386.4	434.4	0.180	1.133	0.073	0.338
17α-Hydroxyprogesterone	11	0.460	0.617	0.382	1.303	0.404	0.667
Androstenedione	11	0.427	0.521	0.334	1.260	0.320	0.667
Aldosterone	11	212.9	208.7	-0.098	0.935	0.660	0.825
Cortisone	11	130.4	131.1	-0.031	0.979	0.832	0.912
Corticosterone	11	15.009	15.289	-0.005	0.997	0.976	0.995
Dehydroepiandrosterone	11	1.444	1.321	0.003	1.002	0.995	0.995
11-Deoxycorticosterone	11	32.246	44.251	0.406	1.325	0.309	0.667
Estrone	11	18.206	30.505	1.066	2.094	0.028	0.272
Cortisol	11	3.515	3.763	0.005	1.003	0.987	0.995
Progesterone	8	0.036	0.060	1.014	2.019	0.123	0.338
11-Deoxycortisol	11	0.488	0.456	-0.301	0.812	0.533	0.707
Testosterone	11	388.6	1646.4	1.553	2.934	0.073	0.338
VA	11	578.3	545.2	-0.091	0.939	0.370	0.667
VD2	11	0.607	1.142	0.432	1.349	0.518	0.707
VD3	11	19.277	24.476	0.319	1.248	0.059	0.338
VE	11	16.549	16.565	-0.020	0.986	0.843	0.912
VK	11	2.322	1.667	-0.522	0.696	0.087	0.338
5-MTHF	11	26.619	28.337	-0.199	0.871	0.721	0.866
VB1	11	2.312	2.080	0.090	1.064	0.790	0.912

Analyte	Paired n	Baseline mean	Week-12 mean	log2 fold change	Fold change	P	q
VB2	11	44.637	40.746	0.172	1.126	0.535	0.707
VB5	11	140.8	106.5	-0.147	0.903	0.675	0.825
VB6PA	11	13.014	13.475	-0.117	0.922	0.829	0.912
Ala	11	449.4	463.2	0.070	1.050	0.673	0.825
Arg	11	47.144	52.486	0.200	1.149	0.343	0.667
Asn	11	49.029	56.605	0.204	1.152	0.093	0.338
Asp	11	3.705	4.064	0.038	1.027	0.924	0.968
Cit	11	38.133	46.807	0.335	1.262	0.114	0.338
Gln	11	522.9	521.9	-0.018	0.988	0.841	0.912
Glu	11	43.720	58.659	0.327	1.255	0.144	0.380
His	11	85.401	88.320	0.048	1.034	0.390	0.667
Ile	11	89.310	94.134	0.099	1.071	0.453	0.704
Leu	11	90.430	94.852	0.072	1.052	0.451	0.704
Lys	11	241.5	240.8	-0.009	0.994	0.910	0.968
Met	11	23.039	24.908	0.123	1.089	0.183	0.432
Orn	11	82.015	92.057	0.173	1.127	0.462	0.704
Phe	11	39.024	43.107	0.152	1.111	0.111	0.338
Pro	11	220.8	275.5	0.308	1.238	0.122	0.338
Ser	11	68.238	79.353	0.205	1.152	0.104	0.338
Thr	11	112.9	119.3	0.102	1.073	0.397	0.667
Trp	11	43.895	47.844	0.094	1.067	0.471	0.704
Tyr	11	66.213	73.319	0.142	1.104	0.109	0.338
Val	11	274.6	285.0	0.056	1.040	0.480	0.704
66 Zn [ppb]	11	1389.1	1286.9	-0.151	0.901	0.215	0.490
55 Mn [ppb]	11	1.213	0.900	-0.415	0.750	0.009	0.192
24 Mg [ppm]	11	19843.6	21249.0	0.096	1.069	0.029	0.272
63 Cu [ppb]	11	882.6	1000.8	0.171	1.126	0.009	0.192
56 Fe [ppb]	11	1101.2	1209.4	0.160	1.117	0.162	0.397
78 Se [ppb]	11	126.9	121.2	-0.059	0.960	0.522	0.707
52 Cr [ppb]	11	0.913	0.782	-0.199	0.871	0.078	0.338
59 Co [ppb]	11	0.165	0.228	0.457	1.372	0.080	0.338
88 Sr [ppb]	11	42.138	46.184	0.126	1.091	0.377	0.667
127 I [ppb]	11	56.699	55.445	-0.039	0.973	0.617	0.799
44 Ca [ppm]	11	15032.2	16264.9	0.112	1.081	0.014	0.238
208 Pb [ppb]	11	1.933	1.965	-0.201	0.870	0.382	0.667
201 Hg [ppb]	10	0.109	0.117	0.049	1.034	0.840	0.912
75 As [ppb]	11	1.513	0.830	-0.539	0.688	0.392	0.667
51 V [ppb]	11	0.479	0.384	-0.337	0.792	0.051	0.338
71 Ga [ppb]	11	0.128	0.098	-1.021	0.493	0.150	0.381

Table S3D. Control: paired molecular changes

Analyte	Paired n	Baseline mean	Week-12 mean	log2 fold change	Fold change	P	q
tHCY	48	12.569	13.223	0.026	1.018	0.681	0.873
Acetate	47	11468.6	11224.4	-0.064	0.956	0.693	0.873
Propionate	47	426.1	494.4	0.531	1.444	<0.001	0.008
Isobutyrate	48	177.2	169.8	-0.010	0.993	0.873	0.918
butyrate	48	455.7	430.0	-0.028	0.981	0.649	0.873
Isovalerate	45	190.0	219.7	0.307	1.238	0.072	0.221
Valerate	45	106.2	99.143	-0.028	0.981	0.891	0.918

Analyte	Paired n	Baseline mean	Week-12 mean	log2 fold change	Fold change	P	q
Caproate	48	402.2	604.6	0.549	1.463	<0.001	<0.001
17α-Hydroxyprogesterone	48	0.815	0.521	-0.732	0.602	<0.001	0.008
Androstenedione	48	0.644	0.544	-0.265	0.832	0.020	0.080
Aldosterone	48	119.9	118.9	0.270	1.206	0.117	0.318
Cortisone	48	80.524	95.807	0.398	1.317	0.006	0.036
Corticosterone	48	11.931	13.093	0.116	1.084	0.465	0.771
Dehydroepiandrosterone	48	1.582	1.742	0.002	1.001	0.993	0.993
11-Deoxycorticosterone	48	30.605	21.678	-0.395	0.760	0.052	0.177
Estrone	48	12.468	20.286	0.204	1.152	0.255	0.558
Estradiol	26	9.142	6.495	0.162	1.119	0.678	0.873
Cortisol	48	78.915	35.576	-1.045	0.485	<0.001	0.008
Progesterone	46	0.061	0.055	0.146	1.107	0.580	0.857
11-Deoxycortisol	48	0.655	0.402	-0.727	0.604	<0.001	0.004
Testosterone	48	1178.8	812.9	-1.356	0.391	0.001	0.008
VA	48	538.3	571.5	0.069	1.049	0.299	0.609
VD2	48	1.335	1.856	0.169	1.124	0.541	0.850
VD3	48	22.744	21.352	-0.052	0.964	0.635	0.873
VE	48	14.421	14.987	0.058	1.041	0.405	0.706
VK	48	1.730	1.555	-0.052	0.965	0.686	0.873
5-MTHF	48	18.558	18.135	0.075	1.053	0.728	0.895
VB1	48	2.081	2.088	0.260	1.197	0.361	0.646
VB2	48	23.261	17.408	-0.250	0.841	0.293	0.609
VB5	48	109.4	109.7	-0.019	0.987	0.856	0.918
VB6PA	48	8.770	8.986	-0.031	0.979	0.871	0.918
Ala	48	365.2	401.4	0.185	1.137	0.026	0.097
Arg	48	64.532	55.322	-0.139	0.908	0.200	0.454
Asn	48	43.997	48.676	0.151	1.110	0.010	0.053
Asp	45	4.573	4.230	-0.156	0.898	0.517	0.837
Cit	48	38.966	44.390	0.175	1.129	0.116	0.318
Gln	48	449.9	511.8	0.215	1.161	<0.001	0.003
Glu	48	46.649	60.150	0.548	1.462	<0.001	0.004
His	48	70.951	74.674	0.046	1.033	0.446	0.757
Ile	48	75.786	74.654	-0.048	0.967	0.563	0.850
Leu	48	96.530	82.662	-0.204	0.868	0.016	0.068
Lys	48	214.6	204.3	-0.104	0.930	0.065	0.211
Met	48	23.223	23.198	0.003	1.002	0.963	0.977
Orn	48	67.787	89.151	0.364	1.287	0.002	0.016
Phe	48	48.677	45.150	-0.062	0.958	0.324	0.630
Pro	48	235.4	188.2	-0.282	0.823	0.014	0.064
Ser	48	70.342	73.334	0.145	1.106	0.104	0.308
Thr	48	108.9	108.0	0.013	1.009	0.886	0.918
Trp	48	42.490	44.831	0.055	1.039	0.305	0.609
Tyr	48	62.589	62.431	-0.019	0.987	0.801	0.912
Val	48	221.3	229.2	0.020	1.014	0.750	0.895
66 Zn [ppb]	48	1136.4	997.9	-0.164	0.892	0.010	0.053
55 Mn [ppb]	28	5.076	0.826	-0.700	0.616	0.014	0.064
24 Mg [ppm]	48	11973.9	12193.3	-0.033	0.977	0.361	0.646
63 Cu [ppb]	48	972.4	1000.7	0.029	1.020	0.685	0.873
56 Fe [ppb]	48	1128.0	1078.3	-0.046	0.968	0.559	0.850
78 Se [ppb]	48	112.2	113.1	-0.028	0.981	0.673	0.873

Analyte	Paired n	Baseline mean	Week-12 mean	log2 fold change	Fold change	P	q
52 Cr [ppb]	47	1.461	0.845	0.092	1.066	0.747	0.895
59 Co [ppb]	30	0.172	0.199	0.260	1.197	0.151	0.366
88 Sr [ppb]	48	45.107	45.832	-0.029	0.980	0.788	0.912
127 I [ppb]	48	48.971	49.154	-0.012	0.991	0.836	0.918
44 Ca [ppm]	48	8602.9	9522.6	0.052	1.037	0.125	0.327
208 Pb [ppb]	28	1.954	1.375	-0.557	0.680	0.001	0.008
201 Hg [ppb]	29	0.105	0.116	-0.055	0.963	0.804	0.912
111 Cd [ppb]	6	0.156	0.093	-1.452	0.366	0.162	0.381
75 As [ppb]	31	1.344	1.386	0.322	1.250	0.346	0.646
51 V [ppb]	37	0.391	0.340	-0.321	0.801	0.047	0.168
71 Ga [ppb]	23	0.125	0.098	-0.514	0.700	0.135	0.339

Table S4. Exploratory baseline-adjusted physical-performance comparisons versus control

Outcome	Contrast	Adjusted difference	95% CI	P	q	Model n
Handgrip strength (kg)	Walking vs Control	0.313	-1.09 to 1.71	0.661	0.661	141
Handgrip strength (kg)	Resistance training vs Control	1.650	0.06 to 3.24	0.042	0.232	141
Handgrip strength (kg)	Traditional Chinese exercise vs Control	-2.053	-4.88 to 0.78	0.155	0.692	141
Back scratch (cm)	Walking vs Control	1.550	-1.53 to 4.63	0.324	0.432	129
Back scratch (cm)	Resistance training vs Control	-1.092	-4.23 to 2.04	0.495	0.620	129
Back scratch (cm)	Traditional Chinese exercise vs Control	-0.439	-3.07 to 2.19	0.743	0.844	129
Chair sit-and-reach (cm)	Walking vs Control	2.188	-1.47 to 5.84	0.241	0.385	135
Chair sit-and-reach (cm)	Resistance training vs Control	3.153	-0.57 to 6.87	0.097	0.257	135
Chair sit-and-reach (cm)	Traditional Chinese exercise vs Control	-0.659	-4.52 to 3.21	0.738	0.844	135
TUG (s)	Walking vs Control	-1.268	-2.23 to -0.31	0.010	0.039	139
TUG (s)	Resistance training vs Control	-1.095	-2.23 to 0.04	0.058	0.232	139
TUG (s)	Traditional Chinese exercise vs Control	0.664	-0.67 to 2.00	0.329	0.844	139
6MWT (m)	Walking vs Control	7.664	-18.38 to 33.70	0.564	0.645	134
6MWT (m)	Resistance training vs Control	-13.406	-56.52 to 29.71	0.542	0.620	134
6MWT (m)	Traditional Chinese exercise vs Control	-3.236	-35.40 to 28.93	0.844	0.844	134
5YWT (s)	Walking vs Control	-0.744	-1.13 to -0.36	<0.001	0.001	141
5YWT (s)	Resistance training vs Control	-0.282	-0.85 to 0.29	0.331	0.529	141
5YWT (s)	Traditional Chinese exercise vs Control	0.470	-0.21 to 1.15	0.173	0.692	141
SPPB (points)	Walking vs Control	0.534	-0.08 to 1.15	0.090	0.180	135
SPPB (points)	Resistance training vs Control	0.002	-0.84 to 0.84	0.996	0.996	135
SPPB (points)	Traditional Chinese exercise vs Control	0.163	-0.59 to 0.92	0.672	0.844	135
Berg balance (points)	Walking vs Control	-0.842	-1.78 to 0.10	0.079	0.180	138
Berg balance (points)	Resistance training vs Control	-0.727	-2.03 to 0.57	0.272	0.529	138
Berg balance (points)	Traditional Chinese exercise vs Control	-0.365	-1.97 to 1.24	0.656	0.844	138

Analysis of covariance modeled week-12 values and adjusted for baseline outcome, age, sex, and BMI, with HC3 robust standard errors. q values were calculated across all eight outcomes separately for each intervention-versus-control contrast. Models were rerun using the corresponding baseline outcome and baseline covariates from the same final dataset used for Tables 1 and S2. Model n includes participants with complete outcome pairs and covariates.

Table S5. Binary frailty transitions and exploratory adjusted risk ratios

Group	Paired n	Robust→robust	Robust→pre-frail/frail	Pre-frail/frail→robust	Pre-frail/frail→pre-frail/frail	Exact McNemar P
Control	52	12	14	4	22	0.031
Resistance training	20	1	1	5	13	0.219
Traditional Chinese exercise	22	5	6	3	8	0.508
Walking	50	9	6	11	24	0.332

Contrast	Adjusted RR	95% CI	P	Model n
Walking vs Control	0.81	0.61 to 1.06	0.118	141
Resistance training vs Control	0.87	0.63 to 1.20	0.389	141
Traditional Chinese exercise vs Control	0.93	0.67 to 1.29	0.665	141

Modified Poisson regression with robust standard errors adjusted for baseline frailty classification, age, sex, and BMI. Control was the reference group. All adjusted frailty intervals include 1. Binary stability does not exclude worsening from pre-frailty to frailty; baseline-stratified proportions and Wilson intervals are in Table S8.

Table S6. Exploratory adjusted molecular comparisons versus control

Table S6A. Hematological measures

Analyte	Contrast	Model n	Adjusted ratio	95% CI	P	q
WBC 10^9/L	Walking vs Control	122	0.978	0.915 to 1.046	0.523	0.560
WBC 10^9/L	Resistance training vs Control	122	0.913	0.822 to 1.014	0.089	0.149
WBC 10^9/L	Traditional Chinese exercise vs Control	122	0.944	0.842 to 1.057	0.317	0.432
Lym# 10^9/L	Walking vs Control	122	0.990	0.875 to 1.121	0.875	0.875
Lym# 10^9/L	Resistance training vs Control	122	0.979	0.829 to 1.157	0.805	0.805
Lym# 10^9/L	Traditional Chinese exercise vs Control	122	0.883	0.766 to 1.019	0.089	0.222
Lym% %	Walking vs Control	122	0.965	0.898 to 1.037	0.330	0.403
Lym% %	Resistance training vs Control	122	1.027	0.870 to 1.212	0.753	0.805
Lym% %	Traditional Chinese exercise vs Control	122	0.908	0.791 to 1.042	0.170	0.284
RBC 10^12/L	Walking vs Control	122	1.026	0.973 to 1.082	0.349	0.403
RBC 10^12/L	Resistance training vs Control	122	1.073	1.010 to 1.139	0.022	0.041
RBC 10^12/L	Traditional Chinese exercise vs Control	122	0.958	0.893 to 1.028	0.237	0.356
HGB g/L	Walking vs Control	122	1.054	1.008 to 1.102	0.022	0.036
HGB g/L	Resistance training vs Control	122	1.035	0.988 to 1.084	0.152	0.228
HGB g/L	Traditional Chinese exercise vs Control	122	0.962	0.914 to 1.012	0.133	0.284
HCT %	Walking vs Control	122	0.049	0.025 to 0.099	<0.001	<0.001
HCT %	Resistance training vs Control	122	5.232	2.463 to 11.114	<0.001	<0.001
HCT %	Traditional Chinese exercise vs Control	122	3.406	1.811 to 6.408	<0.001	0.002
MCV fL	Walking vs Control	122	1.015	1.007 to 1.024	<0.001	0.002

Analyte	Contrast	Model n	Adjusted ratio	95% CI	P	q
MCV fL	Resistance training vs Control	122	1.039	1.028 to 1.051	<0.001	<0.001
MCV fL	Traditional Chinese exercise vs Control	122	0.988	0.980 to 0.997	0.006	0.042
MCH pg	Walking vs Control	122	1.028	1.012 to 1.044	<0.001	0.002
MCH pg	Resistance training vs Control	122	0.954	0.938 to 0.970	<0.001	<0.001
MCH pg	Traditional Chinese exercise vs Control	122	0.998	0.983 to 1.014	0.833	0.833
MCHC g/L	Walking vs Control	122	1.035	1.012 to 1.058	0.002	0.005
MCHC g/L	Resistance training vs Control	122	0.942	0.921 to 0.963	<0.001	<0.001
MCHC g/L	Traditional Chinese exercise vs Control	122	0.998	0.984 to 1.012	0.733	0.785
RDW-CV %	Walking vs Control	122	1.016	1.002 to 1.031	0.027	0.040
RDW-CV %	Resistance training vs Control	122	1.031	1.016 to 1.047	<0.001	<0.001
RDW-CV %	Traditional Chinese exercise vs Control	122	0.979	0.957 to 1.001	0.062	0.222
RDW-SD fL	Walking vs Control	122	1.093	1.026 to 1.164	0.006	0.011
RDW-SD fL	Resistance training vs Control	122	1.094	1.025 to 1.167	0.006	0.016
RDW-SD fL	Traditional Chinese exercise vs Control	122	0.979	0.936 to 1.023	0.346	0.433
PLT 10^9/L	Walking vs Control	122	0.947	0.889 to 1.008	0.087	0.119
PLT 10^9/L	Resistance training vs Control	122	0.975	0.903 to 1.052	0.511	0.697
PLT 10^9/L	Traditional Chinese exercise vs Control	122	1.037	0.941 to 1.141	0.464	0.535
MPV fL	Walking vs Control	122	1.070	1.048 to 1.092	<0.001	<0.001
MPV fL	Resistance training vs Control	122	1.007	0.973 to 1.042	0.696	0.803
MPV fL	Traditional Chinese exercise vs Control	122	0.967	0.934 to 1.001	0.055	0.222
PDW	Walking vs Control	122	0.881	0.835 to 0.930	<0.001	<0.001
PDW	Resistance training vs Control	122	1.056	1.008 to 1.105	0.020	0.041
PDW	Traditional Chinese exercise vs Control	122	1.057	0.992 to 1.127	0.089	0.222
P-LCR %	Walking vs Control	122	1.155	1.088 to 1.225	<0.001	<0.001
P-LCR %	Resistance training vs Control	122	1.027	0.917 to 1.151	0.641	0.802
P-LCR %	Traditional Chinese exercise vs Control	122	0.932	0.846 to 1.028	0.162	0.284

Table S6B. Targeted metabolites

Analyte	Contrast	Model n	Adjusted ratio	95% CI	P	q
tHCY	Walking vs Control	123	1.023	0.905 to 1.156	0.717	0.808
tHCY	Resistance training vs Control	123	1.219	1.038 to 1.431	0.016	0.057
tHCY	Traditional Chinese exercise vs Control	123	0.882	0.700 to 1.111	0.287	0.555
Acetate	Walking vs Control	122	0.505	0.406 to 0.627	<0.001	<0.001
Acetate	Resistance training vs Control	122	1.560	1.204 to 2.021	<0.001	0.004
Acetate	Traditional Chinese exercise vs Control	122	1.443	1.048 to 1.986	0.025	0.109
Propionate	Walking vs Control	121	0.960	0.876 to 1.052	0.382	0.506
Propionate	Resistance training vs Control	121	1.191	1.083 to 1.310	<0.001	0.002
Propionate	Traditional Chinese exercise vs	121	1.121	0.949 to 1.325	0.178	0.441

Analyte	Contrast	Model n	Adjusted ratio	95% CI	P	q
	Control					
Isobutyrate	Walking vs Control	123	0.899	0.828 to 0.977	0.013	0.029
Isobutyrate	Resistance training vs Control	123	0.878	0.808 to 0.954	0.002	0.010
Isobutyrate	Traditional Chinese exercise vs Control	123	1.185	1.050 to 1.338	0.006	0.040
butyrate	Walking vs Control	123	0.905	0.832 to 0.984	0.020	0.042
butyrate	Resistance training vs Control	123	0.920	0.841 to 1.006	0.067	0.167
butyrate	Traditional Chinese exercise vs Control	123	1.232	1.074 to 1.412	0.003	0.025
Isovalerate	Walking vs Control	116	1.111	0.963 to 1.282	0.148	0.241
Isovalerate	Resistance training vs Control	116	0.338	0.276 to 0.414	<0.001	<0.001
Isovalerate	Traditional Chinese exercise vs Control	116	1.213	1.021 to 1.441	0.028	0.117
Valerate	Walking vs Control	116	1.820	1.547 to 2.142	<0.001	<0.001
Valerate	Resistance training vs Control	116	0.380	0.313 to 0.463	<0.001	<0.001
Valerate	Traditional Chinese exercise vs Control	116	1.036	0.776 to 1.383	0.808	0.861
Caproate	Walking vs Control	123	1.370	1.214 to 1.546	<0.001	<0.001
Caproate	Resistance training vs Control	123	1.300	1.132 to 1.493	<0.001	0.002
Caproate	Traditional Chinese exercise vs Control	123	0.746	0.655 to 0.849	<0.001	<0.001
17α-Hydroxyprogesterone	Walking vs Control	123	1.436	0.981 to 2.103	0.063	0.118
17α-Hydroxyprogesterone	Resistance training vs Control	123	0.778	0.355 to 1.708	0.532	0.673
17α-Hydroxyprogesterone	Traditional Chinese exercise vs Control	123	1.259	0.714 to 2.218	0.426	0.690
Androstenedione	Walking vs Control	123	1.199	0.949 to 1.514	0.129	0.222
Androstenedione	Resistance training vs Control	123	0.687	0.360 to 1.310	0.254	0.431
Androstenedione	Traditional Chinese exercise vs Control	123	1.106	0.762 to 1.606	0.596	0.804
Aldosterone	Walking vs Control	123	0.319	0.165 to 0.615	<0.001	0.002
Aldosterone	Resistance training vs Control	123	0.467	0.230 to 0.949	0.035	0.122
Aldosterone	Traditional Chinese exercise vs Control	123	1.074	0.717 to 1.609	0.731	0.839
Cortisone	Walking vs Control	123	0.466	0.317 to 0.685	<0.001	<0.001
Cortisone	Resistance training vs Control	123	2.346	1.187 to 4.636	0.014	0.055
Cortisone	Traditional Chinese exercise vs Control	123	1.050	0.827 to 1.333	0.689	0.839
Corticosterone	Walking vs Control	123	0.536	0.367 to 0.782	0.001	0.004
Corticosterone	Resistance training vs Control	123	1.015	0.387 to 2.658	0.976	0.976
Corticosterone	Traditional Chinese exercise vs Control	123	1.254	0.922 to 1.704	0.149	0.403
Dehydroepiandrosterone	Walking vs Control	123	1.531	1.107 to 2.117	0.010	0.024
Dehydroepiandrosterone	Resistance training vs Control	123	0.388	0.232 to 0.649	<0.001	0.002
Dehydroepiandrosterone	Traditional Chinese exercise vs Control	123	0.825	0.524 to 1.298	0.406	0.690
11-Deoxycorticosterone	Walking vs Control	123	0.892	0.543 to 1.465	0.652	0.777
11-Deoxycorticosterone	Resistance training vs Control	123	0.563	0.319 to 0.993	0.047	0.149
11-Deoxycorticosterone	Traditional Chinese exercise vs Control	123	1.957	1.045 to 3.664	0.036	0.131
Estrone	Walking vs Control	123	0.835	0.516 to 1.350	0.461	0.584
Estrone	Resistance training vs Control	123	0.924	0.560 to 1.522	0.755	0.835

Analyte	Contrast	Model n	Adjusted ratio	95% CI	P	q
Estrone	Traditional Chinese exercise vs Control	123	1.824	0.930 to 3.580	0.080	0.268
Cortisol	Walking vs Control	123	3.157	1.514 to 6.585	0.002	0.006
Cortisol	Resistance training vs Control	123	0.101	0.037 to 0.279	<0.001	<0.001
Cortisol	Traditional Chinese exercise vs Control	123	0.829	0.505 to 1.361	0.458	0.690
Progesterone	Walking vs Control	118	1.957	1.404 to 2.728	<0.001	<0.001
Progesterone	Resistance training vs Control	118	0.770	0.499 to 1.189	0.238	0.422
Progesterone	Traditional Chinese exercise vs Control	118	1.310	0.832 to 2.063	0.243	0.503
11-Deoxycortisol	Walking vs Control	123	1.466	1.108 to 1.940	0.007	0.018
11-Deoxycortisol	Resistance training vs Control	123	0.555	0.288 to 1.073	0.080	0.184
11-Deoxycortisol	Traditional Chinese exercise vs Control	123	1.085	0.639 to 1.840	0.763	0.845
Testosterone	Walking vs Control	123	1.393	0.547 to 3.547	0.487	0.604
Testosterone	Resistance training vs Control	123	0.627	0.199 to 1.977	0.425	0.573
Testosterone	Traditional Chinese exercise vs Control	123	7.032	2.196 to 22.518	0.001	0.016
VA	Walking vs Control	123	1.010	0.898 to 1.135	0.873	0.917
VA	Resistance training vs Control	123	1.453	1.274 to 1.658	<0.001	<0.001
VA	Traditional Chinese exercise vs Control	123	0.913	0.785 to 1.063	0.240	0.503
VD2	Walking vs Control	123	0.995	0.663 to 1.496	0.982	0.982
VD2	Resistance training vs Control	123	0.845	0.544 to 1.315	0.456	0.590
VD2	Traditional Chinese exercise vs Control	123	0.812	0.363 to 1.817	0.612	0.808
VD3	Walking vs Control	123	0.854	0.707 to 1.033	0.104	0.184
VD3	Resistance training vs Control	123	1.588	1.326 to 1.903	<0.001	<0.001
VD3	Traditional Chinese exercise vs Control	123	1.164	0.907 to 1.494	0.231	0.503
VE	Walking vs Control	123	0.730	0.634 to 0.841	<0.001	<0.001
VE	Resistance training vs Control	123	1.026	0.855 to 1.233	0.781	0.835
VE	Traditional Chinese exercise vs Control	123	1.065	0.895 to 1.267	0.481	0.690
VK	Walking vs Control	123	0.572	0.440 to 0.743	<0.001	<0.001
VK	Resistance training vs Control	123	0.867	0.640 to 1.174	0.355	0.524
VK	Traditional Chinese exercise vs Control	123	0.974	0.646 to 1.470	0.901	0.915
5-MTHF	Walking vs Control	123	2.651	1.801 to 3.902	<0.001	<0.001
5-MTHF	Resistance training vs Control	123	0.654	0.401 to 1.065	0.088	0.194
5-MTHF	Traditional Chinese exercise vs Control	123	1.193	0.559 to 2.545	0.648	0.834
VB1	Walking vs Control	123	2.653	1.747 to 4.028	<0.001	<0.001
VB1	Resistance training vs Control	123	1.486	0.807 to 2.737	0.204	0.372
VB1	Traditional Chinese exercise vs Control	123	1.113	0.635 to 1.951	0.708	0.839
VB2	Walking vs Control	123	1.835	1.256 to 2.682	0.002	0.005
VB2	Resistance training vs Control	123	0.849	0.568 to 1.268	0.423	0.573
VB2	Traditional Chinese exercise vs Control	123	1.763	1.046 to 2.969	0.033	0.128
VB5	Walking vs Control	123	1.491	1.227 to 1.812	<0.001	<0.001

Analyte	Contrast	Model n	Adjusted ratio	95% CI	P	q
VB5	Resistance training vs Control	123	0.816	0.622 to 1.069	0.140	0.290
VB5	Traditional Chinese exercise vs Control	123	0.934	0.662 to 1.318	0.699	0.839
VB6PA	Walking vs Control	123	1.927	1.376 to 2.700	<0.001	<0.001
VB6PA	Resistance training vs Control	123	1.013	0.672 to 1.527	0.951	0.967
VB6PA	Traditional Chinese exercise vs Control	123	1.078	0.545 to 2.132	0.829	0.861
Ala	Walking vs Control	123	1.037	0.929 to 1.157	0.515	0.626
Ala	Resistance training vs Control	123	0.926	0.799 to 1.072	0.302	0.480
Ala	Traditional Chinese exercise vs Control	123	1.074	0.862 to 1.338	0.523	0.721
Arg	Walking vs Control	123	0.982	0.870 to 1.108	0.767	0.835
Arg	Resistance training vs Control	123	0.975	0.825 to 1.153	0.771	0.835
Arg	Traditional Chinese exercise vs Control	123	0.963	0.814 to 1.139	0.659	0.834
Asn	Walking vs Control	123	0.915	0.801 to 1.047	0.195	0.303
Asn	Resistance training vs Control	123	0.872	0.759 to 1.002	0.054	0.152
Asn	Traditional Chinese exercise vs Control	123	1.184	1.036 to 1.355	0.014	0.070
Asp	Walking vs Control	120	0.780	0.561 to 1.084	0.139	0.232
Asp	Resistance training vs Control	120	0.574	0.325 to 1.016	0.057	0.153
Asp	Traditional Chinese exercise vs Control	120	0.976	0.612 to 1.556	0.919	0.919
Cit	Walking vs Control	123	0.713	0.607 to 0.836	<0.001	<0.001
Cit	Resistance training vs Control	123	0.761	0.634 to 0.914	0.004	0.016
Cit	Traditional Chinese exercise vs Control	123	1.148	0.932 to 1.414	0.193	0.455
Gln	Walking vs Control	123	1.081	0.996 to 1.174	0.061	0.118
Gln	Resistance training vs Control	123	0.990	0.916 to 1.071	0.811	0.852
Gln	Traditional Chinese exercise vs Control	123	0.957	0.846 to 1.083	0.490	0.690
Glu	Walking vs Control	123	0.969	0.822 to 1.142	0.706	0.808
Glu	Resistance training vs Control	123	1.435	1.210 to 1.703	<0.001	<0.001
Glu	Traditional Chinese exercise vs Control	123	0.949	0.694 to 1.298	0.744	0.839
His	Walking vs Control	123	0.731	0.653 to 0.819	<0.001	<0.001
His	Resistance training vs Control	123	0.926	0.830 to 1.032	0.162	0.314
His	Traditional Chinese exercise vs Control	123	1.158	1.022 to 1.312	0.022	0.104
Ile	Walking vs Control	123	0.738	0.661 to 0.823	<0.001	<0.001
Ile	Resistance training vs Control	123	0.852	0.727 to 0.999	0.048	0.149
Ile	Traditional Chinese exercise vs Control	123	1.320	1.153 to 1.511	<0.001	0.002
Leu	Walking vs Control	123	0.891	0.803 to 0.988	0.029	0.058
Leu	Resistance training vs Control	123	0.875	0.764 to 1.002	0.054	0.152
Leu	Traditional Chinese exercise vs Control	123	1.177	1.049 to 1.322	0.006	0.040
Lys	Walking vs Control	123	0.798	0.714 to 0.891	<0.001	<0.001
Lys	Resistance training vs Control	123	0.896	0.792 to 1.012	0.078	0.184
Lys	Traditional Chinese exercise vs Control	123	1.185	1.043 to 1.347	0.009	0.056

Analyte	Contrast	Model n	Adjusted ratio	95% CI	P	q
Met	Walking vs Control	123	0.907	0.831 to 0.989	0.028	0.057
Met	Resistance training vs Control	123	1.041	0.941 to 1.152	0.435	0.573
Met	Traditional Chinese exercise vs Control	123	1.093	0.987 to 1.211	0.087	0.268
Orn	Walking vs Control	123	0.764	0.635 to 0.919	0.004	0.011
Orn	Resistance training vs Control	123	1.064	0.854 to 1.326	0.580	0.706
Orn	Traditional Chinese exercise vs Control	123	1.053	0.783 to 1.414	0.733	0.839
Phe	Walking vs Control	123	0.986	0.907 to 1.072	0.744	0.823
Phe	Resistance training vs Control	123	1.205	1.085 to 1.339	<0.001	0.003
Phe	Traditional Chinese exercise vs Control	123	1.011	0.914 to 1.118	0.834	0.861
Pro	Walking vs Control	123	1.016	0.863 to 1.197	0.845	0.904
Pro	Resistance training vs Control	123	0.846	0.681 to 1.052	0.133	0.284
Pro	Traditional Chinese exercise vs Control	123	1.505	1.182 to 1.915	<0.001	0.016
Ser	Walking vs Control	123	0.960	0.869 to 1.061	0.426	0.550
Ser	Resistance training vs Control	123	1.021	0.909 to 1.147	0.726	0.833
Ser	Traditional Chinese exercise vs Control	123	1.071	0.902 to 1.272	0.432	0.690
Thr	Walking vs Control	123	0.759	0.680 to 0.847	<0.001	<0.001
Thr	Resistance training vs Control	123	1.033	0.887 to 1.202	0.677	0.807
Thr	Traditional Chinese exercise vs Control	123	1.135	0.968 to 1.331	0.118	0.349
Trp	Walking vs Control	123	0.841	0.762 to 0.928	<0.001	0.002
Trp	Resistance training vs Control	123	0.973	0.848 to 1.116	0.694	0.812
Trp	Traditional Chinese exercise vs Control	123	1.072	0.885 to 1.298	0.476	0.690
Tyr	Walking vs Control	123	0.674	0.615 to 0.739	<0.001	<0.001
Tyr	Resistance training vs Control	123	1.089	0.969 to 1.223	0.151	0.303
Tyr	Traditional Chinese exercise vs Control	123	1.234	1.077 to 1.414	0.003	0.025
Val	Walking vs Control	123	0.753	0.685 to 0.828	<0.001	<0.001
Val	Resistance training vs Control	123	0.809	0.720 to 0.909	<0.001	0.002
Val	Traditional Chinese exercise vs Control	123	1.216	1.071 to 1.381	0.002	0.025
66 Zn [ppb]	Walking vs Control	121	0.891	0.812 to 0.979	0.016	0.035
66 Zn [ppb]	Resistance training vs Control	121	0.888	0.702 to 1.123	0.322	0.487
66 Zn [ppb]	Traditional Chinese exercise vs Control	121	1.239	1.047 to 1.466	0.013	0.070
55 Mn [ppb]	Walking vs Control	42	0.934	0.237 to 3.672	0.922	0.937
55 Mn [ppb]	Resistance training vs Control	42	0.794	0.371 to 1.701	0.553	0.686
55 Mn [ppb]	Traditional Chinese exercise vs Control	42	1.068	0.893 to 1.276	0.471	0.690
24 Mg [ppm]	Walking vs Control	121	1.071	0.943 to 1.217	0.291	0.420
24 Mg [ppm]	Resistance training vs Control	121	0.934	0.791 to 1.103	0.419	0.573
24 Mg [ppm]	Traditional Chinese exercise vs Control	121	1.046	0.961 to 1.140	0.299	0.562
63 Cu [ppb]	Walking vs Control	121	1.081	0.971 to 1.204	0.156	0.248
63 Cu [ppb]	Resistance training vs Control	121	1.061	0.944 to 1.193	0.320	0.487
63 Cu [ppb]	Traditional Chinese exercise vs	121	1.016	0.884 to 1.169	0.821	0.861

Analyte	Contrast	Model n	Adjusted ratio	95% CI	P	q
	Control					
56 Fe [ppb]	Walking vs Control	121	1.134	0.982 to 1.309	0.087	0.159
56 Fe [ppb]	Resistance training vs Control	121	1.196	0.873 to 1.639	0.264	0.431
56 Fe [ppb]	Traditional Chinese exercise vs Control	121	1.123	0.956 to 1.319	0.157	0.405
78 Se [ppb]	Walking vs Control	121	0.943	0.827 to 1.076	0.383	0.506
78 Se [ppb]	Resistance training vs Control	121	0.980	0.865 to 1.110	0.749	0.835
78 Se [ppb]	Traditional Chinese exercise vs Control	121	1.068	0.894 to 1.277	0.467	0.690
52 Cr [ppb]	Walking vs Control	118	0.803	0.558 to 1.156	0.238	0.352
52 Cr [ppb]	Resistance training vs Control	118	2.598	1.313 to 5.141	0.006	0.025
52 Cr [ppb]	Traditional Chinese exercise vs Control	118	1.093	0.877 to 1.362	0.429	0.690
59 Co [ppb]	Walking vs Control	45	0.655	0.090 to 4.746	0.675	0.790
59 Co [ppb]	Resistance training vs Control	45	0.931	0.448 to 1.936	0.848	0.877
59 Co [ppb]	Traditional Chinese exercise vs Control	45	1.353	0.961 to 1.904	0.083	0.268
88 Sr [ppb]	Walking vs Control	121	0.991	0.837 to 1.173	0.912	0.937
88 Sr [ppb]	Resistance training vs Control	121	0.909	0.720 to 1.149	0.426	0.573
88 Sr [ppb]	Traditional Chinese exercise vs Control	121	1.122	0.893 to 1.411	0.323	0.589
127 I [ppb]	Walking vs Control	121	1.076	0.935 to 1.237	0.308	0.430
127 I [ppb]	Resistance training vs Control	121	0.900	0.805 to 1.007	0.067	0.167
127 I [ppb]	Traditional Chinese exercise vs Control	121	1.142	0.953 to 1.368	0.149	0.403
44 Ca [ppm]	Walking vs Control	121	0.815	0.548 to 1.212	0.312	0.430
44 Ca [ppm]	Resistance training vs Control	121	0.678	0.345 to 1.333	0.260	0.431
44 Ca [ppm]	Traditional Chinese exercise vs Control	121	1.137	0.903 to 1.432	0.274	0.548
75 As [ppb]	Walking vs Control	59	0.642	0.316 to 1.306	0.221	0.335
75 As [ppb]	Resistance training vs Control	59	0.276	0.039 to 1.969	0.199	0.372
75 As [ppb]	Traditional Chinese exercise vs Control	59	0.624	0.305 to 1.279	0.198	0.455

Models adjusted for baseline log2 concentration, age, sex, and BMI. These sensitivity analyses may be affected by intervention-timing or analytical-batch confounding and should not be interpreted as causal intervention effects. FDR correction does not remove these sources of confounding and the estimates are not modality-specific biological signatures.

Table S7A. Gut-microbiome diversity analyses

Group	Paired n	Shannon baseline mean	Shannon week-12 mean	Wilcoxon P	PERMANOVA pseudo-F	PERMANOVA P
Walking	39	2.665	2.505	0.146	0.929	0.543
Resistance training	6	2.698	2.594	0.438	0.488	0.270
Traditional Chinese exercise	15	2.639	2.598	0.679	0.231	0.758
Control	42	2.619	2.556	0.233	0.432	0.455

Table S7B. Taxa with nominal paired P < 0.05 before FDR correction

Group	Taxon	Paired n	Mean CLR change	P	q
Control	Allisonella_histaminiformans	42	-0.573	0.047	0.671

Group	Taxon	Paired n	Mean CLR change	P	q
Control	Anaerotignum_lactatifermentans	42	-1.224	0.026	0.540
Control	Bifidobacterium_animalis	42	-0.961	0.031	0.540
Control	Campylobacter_concisus	42	-1.093	0.018	0.540
Control	Dialister_invisus	42	-1.162	0.033	0.540
Control	Enterococcus_avium	42	-0.886	0.034	0.540
Control	Eubacterium_sulci	42	0.592	0.012	0.540
Control	Intestinibacter_bartlettii	42	1.626	0.005	0.323
Control	Lactobacillus_gasseri	42	-0.603	0.032	0.540
Control	Lactococcus_lactis	42	-1.302	0.005	0.323
Control	Streptococcus_sanguinis	42	0.893	0.033	0.540
Control	Veillonella_atypica	42	-1.690	<0.001	0.174
Control	Veillonella_rogosae	42	-1.042	0.035	0.540
Resistance training	Akkermansia_muciniphila	6	-2.067	0.038	0.799
Resistance training	Anaerostipes_hadrus	6	0.650	0.049	0.799
Resistance training	Bacteroides_xylanisolvens	6	-1.218	0.009	0.799
Resistance training	Gordonibacter_pamelaeae	6	0.503	0.023	0.799
Resistance training	Roseburia_intestinalis	6	-3.857	0.025	0.799
Resistance training	Turicibacter_sanguinis	6	-2.903	0.041	0.799
Traditional Chinese exercise	Adlercreutzia_equolifaciens	15	-1.051	0.049	0.873
Traditional Chinese exercise	Bacteroides_ovatus	15	1.251	0.024	0.873
Traditional Chinese exercise	Bifidobacterium_pseudocatenulatum	15	-1.294	0.047	0.873
Traditional Chinese exercise	Clostridium_symbiosum	15	1.870	0.044	0.873
Traditional Chinese exercise	Collinsella_aerofaciens	15	-0.561	0.026	0.873
Traditional Chinese exercise	Dialister_pneumosintes	15	0.988	0.044	0.873
Traditional Chinese exercise	Enterococcus_casseliflavus	15	1.252	0.049	0.873
Traditional Chinese exercise	Streptococcus_gordonii	15	-1.570	0.039	0.873
Traditional Chinese exercise	Turicibacter_sanguinis	15	1.139	0.023	0.873
Walking	Bifidobacterium_bifidum	39	2.229	0.019	0.475
Walking	Clostridium_paraputrificum	39	1.254	0.030	0.475
Walking	Clostridium_scindens	39	0.936	0.029	0.475
Walking	Collinsella_aerofaciens	39	-2.118	0.044	0.630
Walking	Eubacterium_ramulus	39	-2.354	0.009	0.440
Walking	Klebsiella_oxytoca	39	-1.278	0.021	0.475
Walking	Klebsiella_pneumoniae	39	-3.206	0.001	0.221
Walking	Klebsiella_quasipneumoniae	39	-2.728	0.007	0.440
Walking	Klebsiella_variicola	39	-2.973	0.004	0.418
Walking	Peptostreptococcus_stomatis	39	1.209	0.024	0.475
Walking	Prevotella_corporis	39	1.134	0.026	0.475
Walking	Ruminococcus_callidus	39	1.042	0.029	0.475
Walking	Streptococcus_vestibularis	39	-1.562	0.016	0.475
Walking	Weissella_cibaria	39	-1.062	0.046	0.630

Taxa were retained when present in at least 10% of samples, relative abundances were centered-log-ratio transformed, and paired tests were corrected within group using Benjamini–Hochberg. No taxon met $q < 0.05$. PERMANOVA used within-participant label permutations. Failure to detect change is not evidence of an absent response, especially for the six resistance-training pairs. Availability and baseline comparisons are reported in Tables S9A–S9B.

Table S8. Frailty transitions stratified by baseline binary status

Group	Baseline status	Week-12 transition	n/N	%	95% Wilson CI (%)
Walking	Robust	Stable robust	9/15	60.0	35.7 to 80.2
Walking	Robust	Deterioration	6/15	40.0	19.8 to 64.3
Walking	Pre-frail/frail	Improvement to robust	11/35	31.4	18.6 to 48.0
Walking	Pre-frail/frail	Stable pre-frail/frail	24/35	68.6	52.0 to 81.4
Resistance training	Robust	Stable robust	1/2	50.0	9.5 to 90.5
Resistance training	Robust	Deterioration	1/2	50.0	9.5 to 90.5
Resistance training	Pre-frail/frail	Improvement to robust	5/18	27.8	12.5 to 50.9
Resistance training	Pre-frail/frail	Stable pre-frail/frail	13/18	72.2	49.1 to 87.5
Traditional Chinese exercise	Robust	Stable robust	5/11	45.5	21.3 to 72.0
Traditional Chinese exercise	Robust	Deterioration	6/11	54.5	28.0 to 78.7
Traditional Chinese exercise	Pre-frail/frail	Improvement to robust	3/11	27.3	9.7 to 56.6
Traditional Chinese exercise	Pre-frail/frail	Stable pre-frail/frail	8/11	72.7	43.4 to 90.3
Control	Robust	Stable robust	12/26	46.2	28.8 to 64.5
Control	Robust	Deterioration	14/26	53.8	35.5 to 71.2
Control	Pre-frail/frail	Improvement to robust	4/26	15.4	6.2 to 33.5
Control	Pre-frail/frail	Stable pre-frail/frail	22/26	84.6	66.5 to 93.8

Post hoc descriptive sensitivity analysis; N is the number with paired frailty classifications in the indicated baseline stratum. Improvement and deterioration refer only to crossing the robust versus combined pre-frail/frail boundary. Binary stability does not establish unchanged phenotype severity. The sparse initially robust resistance-training stratum (N = 2) is particularly imprecise. These intervals are unadjusted and do not support causal group comparisons.

Table S9A. Baseline characteristics by paired microbiome availability

Group	Availability	n	Age (years)	BMI (kg/m²)	Men, n/N (%)	Pre-frail/frail, n/N (%)
Walking	Paired	39	81.6 ± 5.7	23.5 ± 3.5 (n=39)	11/39 (28.2%)	27/39 (69.2%)
Walking	No pair	11	83.5 ± 6.2	23.0 ± 2.9 (n=11)	3/11 (27.3%)	8/11 (72.7%)
Resistance training	Paired	6	84.8 ± 7.7	21.9 ± 4.0 (n=6)	2/6 (33.3%)	5/6 (83.3%)
Resistance training	No pair	14	80.8 ± 7.1	24.0 ± 3.4 (n=14)	5/14 (35.7%)	13/14 (92.9%)
Traditional Chinese exercise	Paired	15	82.5 ± 5.1	24.5 ± 3.4 (n=15)	6/15 (40.0%)	8/15 (53.3%)
Traditional Chinese exercise	No pair	8	80.0 ± 7.3	26.5 ± 2.2 (n=7)	4/8 (50.0%)	4/8 (50.0%)
Control	Paired	42	82.8 ± 6.5	23.4 ± 3.5 (n=40)	17/42 (40.5%)	18/42 (42.9%)
Control	No pair	10	83.1 ± 6.6	26.0 ± 4.8 (n=10)	6/10 (60.0%)	8/10 (80.0%)

Baseline characteristics are descriptive, not tests of missingness at random. Age and BMI are mean ± SD; observed BMI denominators are shown. Paired profiles require linked, nonzero-abundance profiles at both time points. Participants without pairs include those with one or neither linked profile; these availability patterns do not establish collection or laboratory failure causes.

Table S9B. Microbiome profile linkage patterns in participants without pairs

Group	Baseline only	Week 12 only	Neither time point	Total without pair
Walking	9	2	0	11
Resistance training	13	1	0	14
Traditional Chinese exercise	5	1	2	8
Control	9	1	0	10

Matched against the existing clinical sample identifiers and processed microbiome abundance table. According to the study records, missing microbiome profiles were primarily attributable to participants not providing a stool sample. Missing profiles were not imputed, and the paired-availability analyses used observed linked data only.

Table S10. Availability of selected baseline covariate fields

Field	Walking	Resistance training	Traditional Chinese exercise	Control
MNA-SF nutritional assessment	50/50	20/20	23/23	52/52
MMSE cognitive assessment	50/50	18/20	23/23	51/52
Daily medication count	50/50	18/20	23/23	49/52
Hypertension questionnaire field	50/50	18/20	23/23	49/52

Counts indicate nonmissing stored baseline fields. Individual comorbidity items have the same observed denominators as the hypertension field. MNA-SF assessed nutritional status and MMSE assessed cognitive function; neither was included in the core adjustment set. No medication classes, changes during follow-up, quantified dietary protein/energy intake, or prior structured exercise exposure were incorporated into the core models. These availability counts do not imply adequate control of residual confounding.
